# Transcriptome Profiles Associated to VHSV Infection or DNA Vaccination in Turbot (*Scophthalmus maximus*)

**DOI:** 10.1371/journal.pone.0104509

**Published:** 2014-08-06

**Authors:** Patricia Pereiro, Sonia Dios, Sebastián Boltaña, Julio Coll, Amparo Estepa, Simon Mackenzie, Beatriz Novoa, Antonio Figueras

**Affiliations:** 1 Instituto de Investigaciones Marinas (IIM), CSIC, Vigo, Spain; 2 Institute of Aquaculture, University of Stirling, Stirling, Scotland, United Kingdom; 3 Dpto Biotecnología, Instituto Nacional Investigaciones Agrarias (INIA), Madrid, Spain; 4 Instituto de Biología Molecular y Celular (IBMC), Miguel Hernández University, Elche, Spain; 5 Institut de Biotecnologia i de Biomedicina, Universitat Autònoma de Barcelona, Barcelona, Spain; University of California Riverside, United States of America

## Abstract

DNA vaccines encoding the viral G glycoprotein show the most successful protection capability against fish rhabdoviruses. Nowadays, the molecular mechanisms underlying the protective response remain still poorly understood. With the aim of shedding light on the protection conferred by the DNA vaccines based in the G glycoprotein of viral haemorrhagic septicaemia virus (VHSV) in turbot (*Scophthalmus maximus*) we have used a specific microarray highly enriched in antiviral sequences to carry out the transcriptomic study associated to VHSV DNA vaccination/infection. The differential gene expression pattern in response to empty plasmid (pMCV1.4) and DNA vaccine (pMCV1.4-G_860_) intramuscular administration with regard to non-stimulated turbot was analyzed in head kidney at 8, 24 and 72 hours post-vaccination. Moreover, the effect of VHSV infection one month after immunization was also analyzed in vaccinated and non-vaccinated fish at the same time points. Genes implicated in the Toll-like receptor signalling pathway, IFN inducible/regulatory proteins, numerous sequences implicated in apoptosis and cytotoxic pathways, MHC class I antigens, as well as complement and coagulation cascades among others were analyzed in the different experimental groups. Fish receiving the pMCV1.4-G_860_ vaccine showed transcriptomic patterns very different to the ones observed in pMCV1.4-injected turbot after 72 h. On the other hand, VHSV challenge in vaccinated and non-vaccinated turbot induced a highly different response at the transcriptome level, indicating a very relevant role of the acquired immunity in vaccinated fish able to alter the typical innate immune response profile observed in non-vaccinated individuals. This exhaustive transcriptome study will serve as a complete overview for a better understanding of the crosstalk between the innate and adaptive immune response in fish after viral infection/vaccination. Moreover, it provides interesting clues about molecules with a potential use as vaccine adjuvants, antiviral treatments or markers for vaccine efficiency monitoring.

## Introduction

Viral haemorrhagic septicaemia virus (VHSV) is a fish pathogen belonging to the genus *Novirhabdovirus*, within the family *Rhabdoviridae*. This etiological agent causes an important viral disease affecting rainbow trout *Oncorhyncus mykiss* and other salmonids [1]–[3] but VHSV outbreaks have been detected in other marine farmed fish species such as turbot (*Scophthalmus maximus* L. 1758) [4], [5]. Nowadays, the culture of this flatfish is well-established being a very important commercial species for the aquaculture industry in Europe and Asia. However, infectious diseases are one of the most relevant limiting factors, causing severe economic losses in many cases. Neither vaccines nor therapeutic treatments are commercially available for this disease. Increased efforts were performed for more than 30 years in order to produce an efficient, safe and cost-effective vaccine against VHSV using subunits or single viral proteins as well as killed or attenuated viruses [6]–[11]. Although some of those vaccines have induced good protection levels in laboratory conditions, they can either be unsafe for field use, its production very expensive or require high doses. DNA vaccination is based on the administration of a plasmidic DNA vector containing the gene encoding a specific antigen. This technology is a powerful tool for the design of effective vaccines against fish rhabdoviral pathogens. Rhabdoviruses possess a surface glycoprotein G that acts as the target of virus neutralizing antibodies [12] and therefore, the most successful DNA vaccines against these viruses are based on the G glycoprotein gene under the control of the cytomegalovirus promoter (CMV). We have recently constructed a DNA vaccine encoding the G glycoprotein from VHSV strain UK-860/94 (isolated from infected turbot) and have demonstrated the high degree of protection provided against this virus as well as the production of specific neutralizing antibodies one month after vaccination [13]. However, the early immune mechanisms implicated in the success of that vaccination remain still unclear.

Microarray technology is a very useful tool for the understanding of the immune process implicated in the protective response provided by efficient DNA vaccines against fish rhabdoviral infection. Some studies have been previously performed using microarrays, including the effect of a DNA vaccine encoding the infectious hematopoietic necrosis virus (IHNV) G glycoprotein in trout [14], the effect of the expression of the G protein from VHSV in Japanese flounder [15], [16], as well as the differences in the gene expression profile following hirame rhabdovirus (HIRRV) G and N protein DNA vaccination in Japanese flounder [17] and the expression pattern after HIRRV challenge in vaccinated and non-vaccinated fish [18]. However, the information provided by these reports was, in some cases, limited due to the relatively low number of annotated immune-related sequences included in the microarray. To our knowledge, this is the first global work in fish including both the analysis of the expression profile after DNA vaccination and the analysis of the differential transcriptomic patterns in vaccinated and non-vaccinated fish after rhabdoviral infection and the first performed in turbot. Moreover, the microarray has been constructed using a high number of annotated sequences obtained from an enriched 454-pyrosequencing of turbot transcriptome after viral stimulations [19], providing a higher quantity of information compared to previous similar publications in other fish species. The gene expression patterns of several immune-relevant pathways were analyzed and allowed a better comprehension of the protective mechanisms underlying VHSV G protein DNA vaccination before and after VHSV challenge.

## Materials and Methods

### Ethical statement

Experimental procedures followed Spanish Law (Royal Executive Order, 53/2013) for Animal Experimentation, in accordance with European Union directive 2010/63/UE. Fish care and challenge experiments were reviewed and approved by the CSIC National Committee on Bioethics (approval number: 07_09032012).

### Fish

Juvenile turbot (average weight 2.5 g) were obtained from a VHSV-free commercial fish farm (Insuiña S.L., Mougás, Galicia, Spain). Animals were maintained in 500 L fibreglass tanks at the IIM (CSIC) facilities with a re-circulating saline water system with a light-dark cycle of 12∶12 h at 18°C and fed daily with a commercial diet (LARVIVA-BioMar). Prior to experiments, fish were acclimatized to laboratory conditions for 2 weeks.

### Plasmids

The expression vector pMCV1.4 (Ready-Vector, Madrid, Spain) was used for the construction of the vaccine containing the G glycoprotein cDNA sequence from VHSV strain UK-860/04 (GeneBank accession number AY546628) as was previously described by Pereiro et al. [13]. High amounts of DNA vaccine (pMCV1.4-G_860_) and the corresponding empty plasmid (pMCV1.4) were obtained by transformation in One Shot TOP10F’ chemically competent *E. coli* (Invitrogen) for its cloning following the protocol instructions. Bacteria were cultured on LB-Kanamycin (50 µg/ml) agar plates during 24 h at 37°C and an isolated colony from each transformation was selected and cultured in LB-Kanamycin (50 µg/ml) medium at 37°C in agitation. Plasmid constructions were purified using the PureLink HiPure Plasmid Midiprep Kit (Invitrogen) following the manufacturer’s instruction.

### VHSV strain UK-860/94

Viral haemorrhagic septicaemia virus UK-860/94 (VHSV_860_) isolated from farmed turbot in Scotland [5] was propagated in Epithelioma papulosum cyprini (EPC) cells at 14°C containing Eagle’s minimum essential medium (MEM, Gibco) supplemented with 2% fetal bovine serum (FBS), penicillin (100 IU/ml) (Invitrogen) and streptomycin (100 mg/ml) (Invitrogen). The supernatants were clarified by centrifugation at 4000×g during 30 min and viruses from these supernatants were concentrated by ultracentrifugation at 100,000×g for 45 min. VHSV_860_ aliquots were maintained at –80°C until use. Virus stock was titrated in 96-well plates according to Reed & Muënch [20] and the *in vivo* infectivity was tested using juvenile turbot.

### Immunization and viral infection protocols

**Figure 1:**
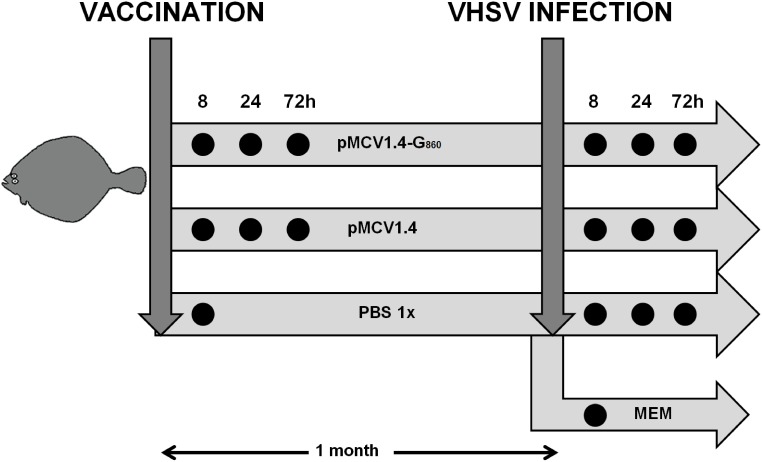
Diagram reflecting the experimental design and sampling points employed in the study of the transcriptomic profiles associated to the vaccination and VHSV infection. This experimental prodecure is explained in detail in the materials and methods section.

A schematic overview of the vaccination/challenge protocol and sampling procedure is shown in Fig. 1. A total number of 204 juvenile turbot were divided into 3 groups, two of them containing 72 fish and the last one 60 fish. Turbot were anaesthetized by immersion in 50 mg/ml buffered tricaine methanesulfonate (MS-222; Sigma) and then, fish from the first two groups were intramuscularly (i.m.) injected with 50 µl of PBS containing 2 µg of pMCV1.4 or pMCV1.4-G_860_. Turbot from the last batch were i.m. inoculated with 50 µl of PBS. At 8, 24 and 72 h after injection, 12 fish were removed from the first two tanks and, at 8 h after PBS inoculation, other 12 fish were taken from the last tank. These turbot were sacrificed by anaesthetic overdose and the head kidney was removed. Equal amounts of tissue from three fish belonging to the same tank and sampling point were pooled, obtaining 4 biological replicates for each treatment and time point (3 turbot/replicate). The remaining fish (36 in the plasmid-injected groups and 48 in the PBS-inoculated tank) were maintained during one month and then, 12 fish from the PBS injected group were separated to another tank. This new group of fish was intraperitoneally (i.p.) injected with 50 µl of MEM + penicillin and streptomycin+2% FBS (PBS - MEM group), whereas the other turbot were i.p. infected with a dose of VHSV_860_ of 5×10^5^ TCID_50_/fish (pMCV1.4 - VHSV and pMCV1.4-G_860_ - VHSV groups). At 8, 24 and 72 hours after infection, 12 fish were removed from the VHSV-infected tanks, and at 8 h after MEM injection the 12 fish were taken from the non-infected tank. The fish were sacrificed by anaesthetic overdose and the head kidney was removed. Equal amounts of tissue from three fish belonging to the same tank and sampling point were pooled, obtaining 4 biological replicates for treatment and time point (3 turbot/replicate). The whole experimental procedure was conducted in parallel to the previously published work in which the high protective effect induced by the pMCV 1.4-G_860_ vaccine was demonstrated, obtaining a relative percent survival (RPS) higher than 80% [13]. Mortality events were not recorded in the absolute control groups (non-immunized and non-infected fish) during the experiment. The health status of the fish was daily monitored and no adverse health effects were observed in non-infected turbot. The animals used in this work were sacrificed before they exhibited clinical signs of disease (8, 24 and 72 hours post-infection).

### Total RNA extraction, quality control and cDNA synthesis

RNA was extracted from 68 samples using TRIzol (Invitrogen) in accordance with instructions provided by the manufacturer in combination with the RNeasy mini kit (Qiagen) for RNA purification after DNase treatment (RNase-free DNase set, Qiagen). RNA concentration was quantified using the spectrophotometer Nanodrop ND-1000 (Thermo Scientific). RNA integrity and quality were also assessed (Bioanalyzer 2100, Agilent Technologies). The RNA integrity number (RIN) was calculated for each sample and only RNAs with a RIN number >7 were processed. For qPCR validation of the microarray results, the cDNA synthesis was performed with SuperScript II Reverse Transcriptase (Invitrogen) using 0.5 µg of RNA by following the manufacturer indications.

### Oligonucleotide microarray design

A specific turbot microarray enriched in immune-related genes was designed by selecting the sequences obtained after a 454-pyrosequencing of several *S. maximus* tissues at different sampling points after viral stimulation (VHSV strain UK-860/94 and Nodavirus strain AH95-NorA) or using molecules mimicking viral infection (pMCV1.4, pMCV1.4-G_860_ and Poly I:C) [19]. This tool was used for analyzing the transcriptome profiles associated to the VHSV infection or DNA vaccination in turbot, as well as the differences between vaccinated and non-vaccinated fish after viral infection. The five proteins encoded by the VHSV genome (RNA-directed RNA polymerase L, Nucleoprotein, Phosphoprotein, Spike glycoprotein and Matrix protein), whose sequences were obtained in the pyrosequencing, were also included in the microarray in order to analyze the evolution of the viral replication in vaccinated and non-vaccinated fish.

A total of 43,398 oligonucleotide probes (60-mer long each) were used to construct a high-density turbot microarray based on the Agilent 4×44K design format using the Agilent eArray interface. Thus, 10,907 annotated contigs were spotted in triplicated into the slide (total probes 32,721), as well as 4,654 spotted in duplicated (9,308). In addition, 18 selected singletons with 2 replicated probes (total probes 36) were also spotted as well as 1,417 internal control probes of Agilent (N = 43,398). The microarray platform TurbotV2_SSFN (ID041183 Agilent) has been submitted to the Gene Expression Omnibus (GEO) repository under accession number GPL16776.

### RNA Labeling and Microarray Hybridization

RNA labelling, hybridizations and scanning were performed according to manufacturer’s instructions. Total RNA (500 ng) was amplified and Cy3-labeled with Agilent’s One-Color Microarray-Based Gene Expression Analysis (Quick Amp Labelling kit) along with Agilent’s One-Color RNA SpikeIn Kit. Each amplified and labelled sample was briefly hybridized at 65°C for 17 hours. Microarray slides were scanned with Agilent Technologies Scanner model G2505B. Spot intensities and other quality control features were extracted with the Feature Extraction software version 10.4.0.0 (Agilent). One-channel TIFF images were imported into the GeneSpring GX 12.0 software (Agilent).

### Microarray data analysis

Fluorescence intensity data and quality measurements were analyzed using the GeneSpring GX 12.0 software (Agilent). After grouping the biological replicates (4 replicates for treatment and sampling point), data were filtered by flags and then by expression between the 20 and 95^th^ percentile in the raw data. Once the analysis by probes was performed, the gene-level experiment was conducted normalizing the data by percentile shifts at the 75^th^ percentile and using as baseline transformation the median of all samples. In order to identify differentially expressed genes, the normalized data were analyzed by filtering on Volcano Plot in order to compare the mean expression levels between treatments (pMCV1.4 and pMCV1.4-G_860_ treatments against PBS [8 h] -control group- and PBS - VHSV, pMCV1.4 - VHSV and pMCV1.4-G_860_ - VHSV treatments against PBS - MEM [8 h] -control group-). An unpaired t-test was conducted without correction and data were considered significant at p<0.05. The fold-change cut-off was set at 1.5. The data presented in this publication and the MIAME-compliant information has been deposited in the NCBI’s Gene Expression Omnibus (GEO, http://www.ncbi.nlm.nih.gov/geo/) and is available under the accession number GSE56487.

The five viral proteins encoded by VHSV were excluded for subsequent analysis of the turbot transcriptomic profiles. Venn diagrams representing the number of shared and exclusive modulated genes among different experimental conditions were also constructed by using GeneSpring GX 12.0 software. This bioinformatic tool was also chosen for performing hierarchical clustering using Euclidean distance metric of several groups of selected genes. Blast2GO suite [21] was used for Gene Ontology (GO) classification into biological process terms of the significantly modulated genes from each comparison.

### qPCR validation

The expression profiles of five immune-related genes modulated in the microarray (Tumor necrosis factor, Interferon phi 2, Interferon-induced GTP-binding protein Mx, IFI56 and Interferon-stimulated gene 15) were determined at 3 different times from vaccinated and non-vaccinated fish by using reverse transcriptase real-time quantitative qPCR. Specific PCR primers were designed using the Primer3 program [22] and their amplification efficiency was calculated using seven serial five-fold dilutions of head kidney cDNA from unstimulated turbot with the Threshold Cycle (C_T_) slope method [23]. Primer sequences are listed in the Table S1. Individual reactions were carried out in 25 µl reaction volume using 12.5 µl of SYBR GREEN PCR Master Mix (Applied Biosystems), 10.5 µl of ultrapure water (Sigma-Aldrich), 0.5 µl of each specific primer (10 µM) and 1 µl of five-fold diluted cDNA template in MicroAmp optical 96-well reaction plates (Applied Biosystems). All reactions were performed using technical triplicates in a 7300 Real-Time PCR System thermocycler (Applied Biosystems) with an initial denaturation (95°C, 10 min) followed by 40 cycles of a denaturation step (95°C, 15 s) and one hybridization-elongation step (60°C, 1 min). An analysis of melting curves was performed for each reaction. Relative expression of each gene was normalized using the Elongation Factor-1 alpha as reference gene, which was constitutively expressed and not affected by the experimental treatments, and calculated using the Pfaffl method [23]. The correlation between microarray and qPCR data (Log_10_ fold-change) were analyzed by the Spearman’s Rho test.

## Results and Discussion

### Validation of microarray data by qPCR

**Figure 2:**
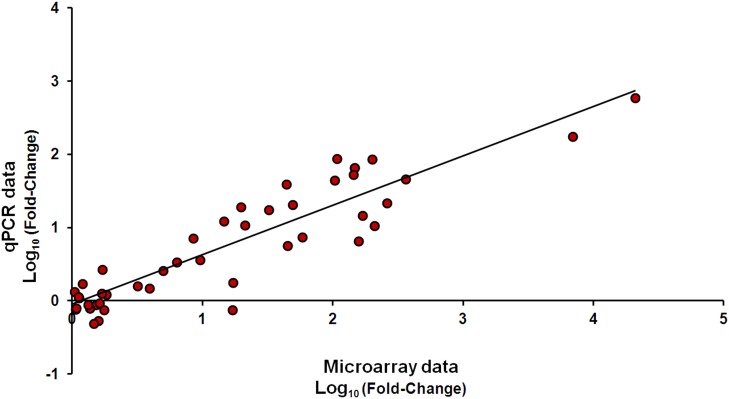
qPCR validation of the microarray data. Correlation between microarray (x-axis) and qPCR (y-axis) data (Log_10_ fold-change) from 5 genes at 3 different times after infection of vaccinated and unvaccinated turbots. Fold-change values of the selected genes were displayed in Table S1. The correlation between microarray and qPCR data analyzed by the Spearman’s Rho test was ρ = 0.969 and with a statistical significance of p<0.01.

Quantitative real-time PCR (qPCR) is a commonly used validation tool for confirming gene expression results obtained from microarray analysis [24]. In order to validate the expression profiles from microarray analysis, the relative mRNA level for 5 immune-relevant genes (Tumor necrosis factor, Interferon phi 2, Interferon-induced GTP-binding protein Mx, IFI56 and Interferon-stimulated gene 15) was measured by qPCR. The expression data obtained by microarray and qPCR for the selected genes are listed in the Table S2 and plotted in Fig. 2. Microarray and qPCR results were analyzed by Spearmańs Rho test and a high correlation (ρ = 0.969) and a statistical significance (p<0.01) were observed.

### Transcription of viral genes in non-vaccinated and vaccinated turbot infected with VHSV

**Figure 3:**
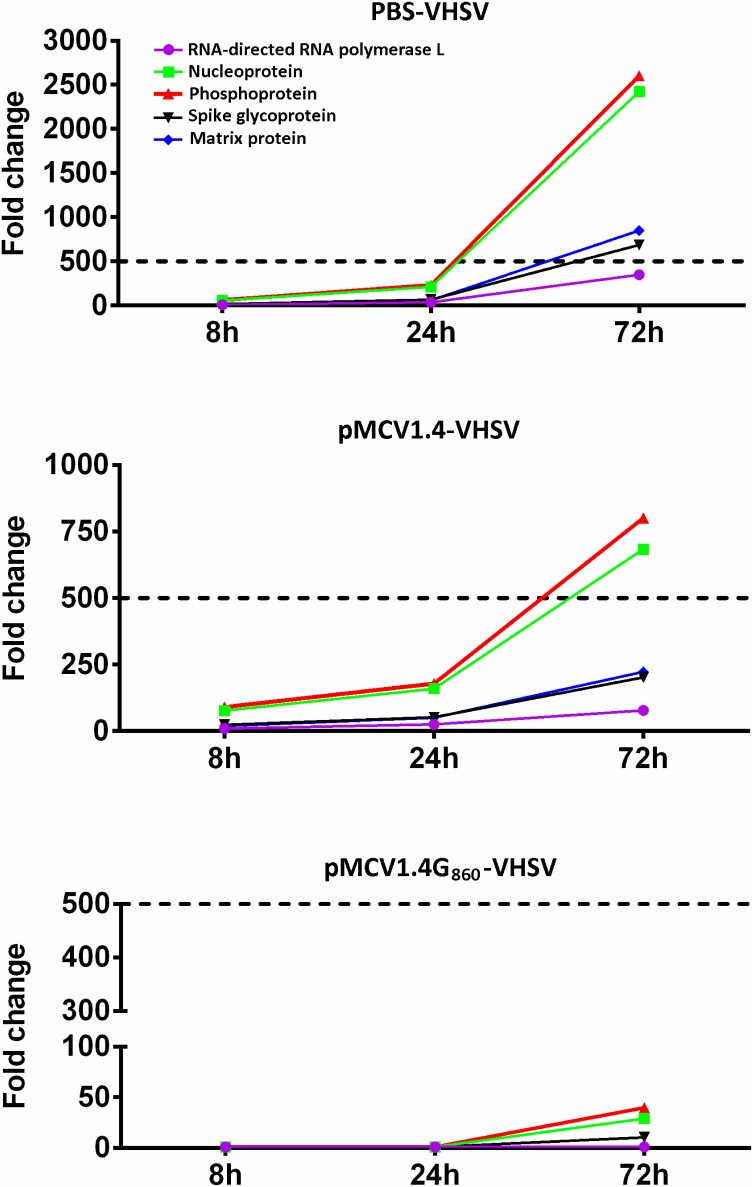
Evolution of viral genes transcription in non-vaccinated and vaccinated turbot. Sequences for the five proteins encoded by VHSV were included in the microarray design for analyzing their evolution in non-vaccinated (PBS – VHSV and pMCV1.4– VHSV) and vaccinated (pMCV1.4-G_860_– VHSV) individuals at 8, 24 and 72 h post h post-challenge.

In order to assess the viral replication success of VHSV strain UK-860/94 in head kidney cells from vaccinated and non-vaccinated fish infected with VHSV, the expression of the five viral genes was analyzed. The evolution of the VHSV replication throughout the tested sampling points (8, 24 and 72 hours after infection) in the different experimental groups is shown in Fig. 3. Those fish that were previously inoculated intramuscularly with PBS or with the empty plasmid (pMCV1.4) and one month later infected with VHSV showed an increasing viral replication from 8 hours to 72 hours post-challenge, being the five proteins already detected at 8 hours. On the other hand, turbot previously injected with the DNA vaccine encoding the G glycoprotein (pMCV1.4-G_860_) showed a limited quantity of viral transcripts after VHSV challenge. Thus, matrix protein and RNA-directed RNA polymerase L were not significantly increased in vaccinated fish at any of the tested time points, whereas the other three genes were only detected after 72 hours post-infection and in a lower level in comparison with the other two groups of non-vaccinated fish. This reduction in the number of viral transcripts in the host cells and, as consequence, the high survival rates obtained after vaccination, might be directly related to the presence of specific neutralizing antibodies against VHSV (strain UK-860/94) one month after immunization as was previously reported [13]. However, we cannot rule out that some non-specific immune responses could also be contributing to the reduction of viral replication because it is known that the specific protection provided by VHSV and IHNV G gene DNA vaccines in fish is preceded by a protective nonspecific antiviral response, possibly related to interferon-induced mechanisms [25]. In fact, interestingly, turbot previously receiving only the empty plasmid and then challenged with VHSV one month later showed a significantly reduced expression of all VHSV genes when compared to the PBS-injected VHSV-challenged group. This reduction in the transcription of viral genes could be related to the persistence of a non-specific immune response probably due to the induction of several immune factors by unmethylated CpG motifs present in the plasmid backbone [26]–[28]. DNA vaccines are constructed from plasmids of bacterial DNA that are able to induce the maturation, differentiation and proliferation of the immune cells and, therefore, to increase the production of the several cytokines [29]. Ritter et al. [30] revealed that immunization using the empty plasmids pcDNA3 and pORF was able to reduce the number of colony-forming units (CFU) of the bacteria *Paracoccidioides brasiliensis* in mice. Other publication showed some but modest protective effect against *Mycobacterium tuberculosis* in mice after vaccination with the empty plasmid pGX10 [31]. With regard to fish viruses, a protective response against the Infectious Pancreatic Necrosis Virus (IPNV) in Atlantic salmon was also observed one week after CpG oligodeoxynucleotides stimulation, revealing the induction of a nonspecific immune response against virus [32]. Indeed, some experiments have shown that the early immune response induced shortly after DNA vaccination against VHSV in trout is non-specific and cross-protective against other rhabdoviruses [33], [34] and even against nodavirus in turbot [35]. Although in our turbot vaccination trials we did not observe any significant difference in final mortalities between PBS and empty plasmid-injected fish groups, only a slight reduction and delay in the mortality [13], our results suggest the importance of the intrinsic adjuvant properties of the plasmids used in DNA vaccination [28] and the persistence of the non-specific immune response.

### Overall effect of pMCV1.4-G_860_ immunization on the host gene expression

**Figure 4:**
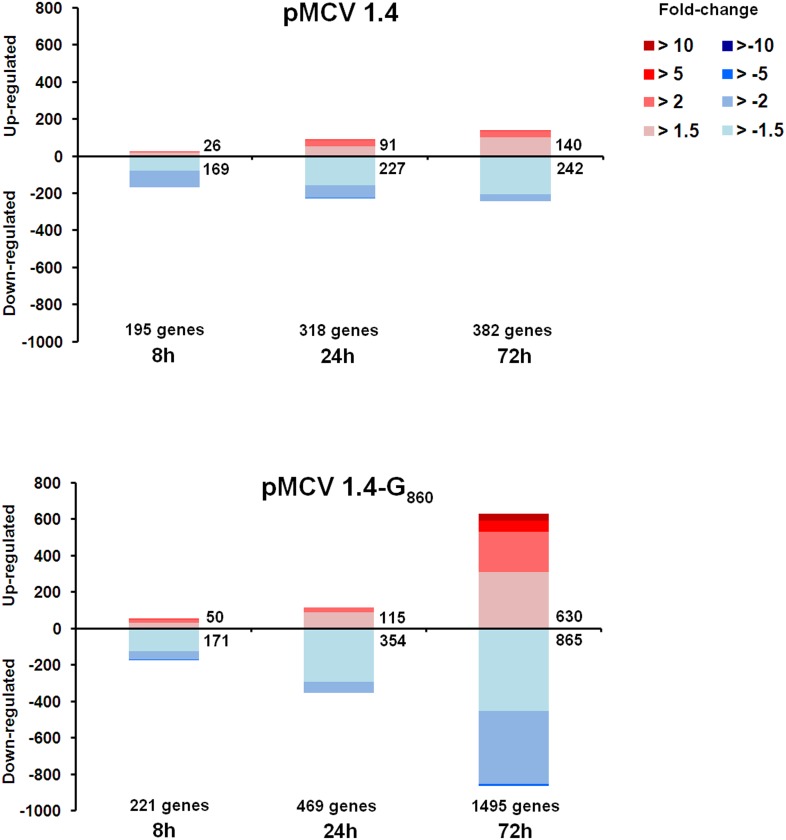
Stacked column chart reflecting the distribution of regulated genes through time after pMCV1.4 or pMCV1.4-G_860_ intramuscular injection. Statistically significant differential gene expressions are subdivided according to intensity (fold change) and sense (up and down-regulation).

Total quantity and fold-changes of up and down-regulated genes in head kidney after pMCV1.4 or pMCV1.4-G_860_ intramuscular injections are shown as stacked column charts in Fig. 4. The number of modulated genes increased with time when compared to the control group (PBS-injected fish) in both cases. In agreement with the reduction of the viral genes expression, the empty plasmid was able to induce the modulation of several genes and this induction increased from 8 to 72 h. Differences in the number of modulated genes between pMCV1.4 and pMCV1.4-G_860_ were highest at 72 h, when the transcription of the G glycoprotein gene is on-going as was previously observed [13]. Intramuscular administration of microgram amounts of DNA vaccine is enough for the expression of the viral G glycoprotein on the surface of muscular cells and this is the way to trigger the orchestration of an adaptive immune response [25], [36]. A total of 1,495 genes were found to be regulated at 72 h after vaccination (630 up-regulated and 865 down-regulated), whereas the empty plasmid induced the expression of 382 genes at the same time point (140 up-regulated and 242 down-regulated).

A Gene Ontology (GO) classification of biological processes at the 2^nd^ level of the modulated genes after plasmids injection is provided in Fig. S1. Those GO categories containing a high representation of genes with a direct implication in immunity (Viral reproduction, Signaling, Response to stimulus, Death, Cell proliferation and Immune system process) showed, in general, their highest representations at 24 h when the empty plasmid was administrated and at 72 h after pMCV1.4-G_860_ vaccination.

Venn diagrams representing exclusive and common genes after pMCV1.4 or pMCV1.4-G_860_ injection are provided in Fig. S2. The number of exclusive genes at 72 h after vaccination was 580 for the up-regulated sequences and 799 for the down-regulated sequences, whereas the empty plasmid administration induced the up-regulation of 90 exclusive genes and the down-regulation of 176 at 72 h. In order to identify those GO categories especially affected during the expression of the G glycoprotein, biological process multilevel pie charts of exclusive and common regulated sequences at 72 h after DNA vaccine or pMCV1.4 empty plasmid injection were constructed and are displayed in Fig. S3. Several immune-related GO terms were found to be highly represented among the pMCV1.4-G_860_ exclusive genes, such as I-kappaB kinase/NF-kappaB cascade, activation of immune response, antigen processing and presentation, cytokine production, immune effector process, innate immune response and regulation of apoptosis, among others. Proteolysis was found to be the category including the highest number of exclusive sequences both in the group of up and down-regulated genes (29 and 34 genes, respectively), which is a GO term containing several molecules implicated in the antigen processing and presentation. On the other hand, empty plasmid exclusive genes as well as common genes between both groups did not show a remarkable representation of these terms at the tested time point.

### Differential gene expression profile in vaccinated and non-vaccinated turbot after VHSV challenge

**Figure 5:**
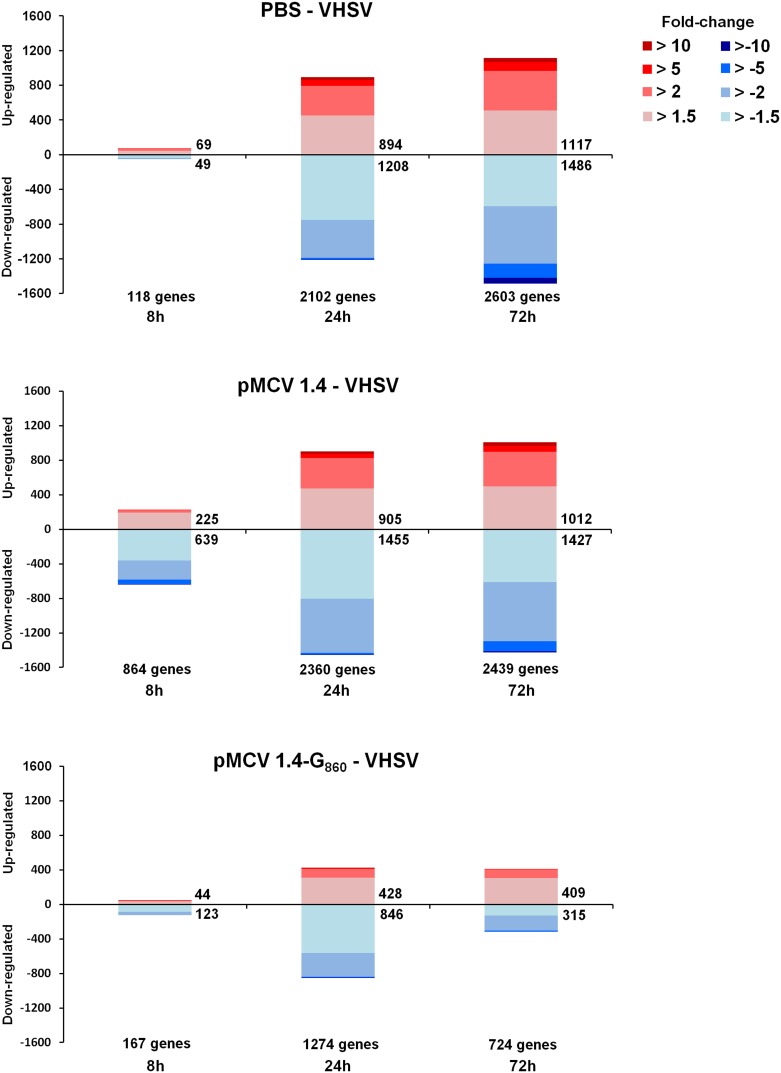
Stacked column chart reflecting the distribution of regulated genes through time after VHSV challenge in non-vaccinated (PBS – VHSV or pMCV1.4– VHSV) and vaccinated (pMCV1.4-G_860_– VHSV) turbot. Statistically significant differential gene expressions are subdivided according to intensity (fold change) and sense (up and down-regulation).

Stacked column charts reflecting the number and intensity of up and down-modulated genes in non-vaccinated and vaccinated fish after VHSV challenge is represented in Fig. 5. The pattern of regulated genes induced by VHSV challenge is quite different from that observed after viral infection in vaccinated fish. The viral challenge induced a time-increasing modulation in the global gene expression, whereas vaccinated fish showed the highest number of up and down-regulated genes at 24 h. Moreover, the number of genes appearing significantly modulated in vaccinated individuals at 24 and 72 h after VHSV infection is quite lower in comparison with the other treatments. Another remarkable point is the difference observed between both non-vaccinated groups (PBS – VHSV and pMCV1.4– VHSV), where the fish previously receiving the empty plasmid showed a higher number of modulated genes at 8 h, suggesting once again a persistence of innate immune factors one month after empty plasmid injection.

A GO classification of biological processes at the 2nd level (Fig. S4) revealed a very higher number of those sequences with direct implication in immune defense in the pMCV1.4– VHSV group at 8 h compared to the PBS – VHSV fish. Unlike the other two treatments, where the number of immune genes increased from 8 to 72 h, vaccinated individuals showed the peak of maximum expression of these sequences at 24 h and a relevant reduction at 72 h. But, interestingly, as it was illustrated in the Venn diagrams (Fig. S5) vaccinated fish presented the major number of exclusive modulated genes at 72 h. Therefore, although the number of regulated sequences decreased at 72 h and the total number of up and down-regulated genes was lower in vaccinated fish with regard to the other groups, this exclusivity could be associated with genes directly related with the existence of adaptive immunity.

### Hierarchical clustering analysis of pathways or groups of molecules involved in defense mechanisms

In order to effectively combat viral infections and other diseases, vertebrate organisms have developed an efficient, powerful and integrated defense network comprising both innate and adaptive immune mechanisms. Numerous defensive processes or families of molecules implicated in non-specific or specific responses against VHSV were analyzed using hierarchical clustering in order to define the transcriptomic profiles after pMCV1.4-G_860_ vaccination as well as after VHSV infection in vaccinated and non-vaccinated turbot. Sequences directly related with the TLR pathway, IFN system, apoptosis, MHC-I antigen presentation, and coagulation among others were shown to be involved in the viral infection and also in the protection provided by the vaccine.

#### TLR pathway

Virus detection by the innate immune system is carried out by a class of molecules known as pattern recognition receptors (PRRs), which detect specific evolutionary conserved structures on pathogens, termed pathogen-associated molecular patterns (PAMPs) [37]. Toll-like receptors (TLRs), a class of PRRs, have been established to play a crucial role in the innate immune response to pathogens through the activation of intracellular signalling pathways, which ultimately induce expression of a large number of genes encoding type I interferons (IFNs), inflammatory cytokines and chemokines, and other molecules affecting the initiation of adaptive immune responses [38].

**Figure 6:**
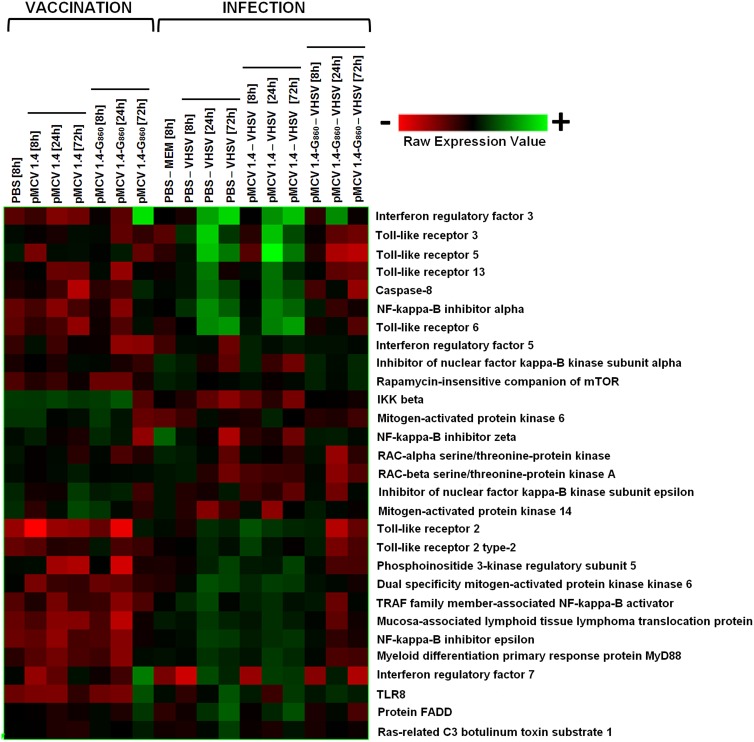
Heat map displaying hierarchical clustering results from microarray expression data of genes implicated in the Toll-like receptor signalling pathway. All the experimental groups, including the corresponding controls (PBS 8 h and PBS – MEM 8 h), were included in the analysis. Different genes are represented in different rows, and different experiments in different columns. Raw expression values are represented as a color scale from red for lower expressions to green for higher expressions.

Six different TLRs were found to be modulated by some treatment in the microarray analysis (TLR2, TLR3, TLR5, TLR6, TLR8 and TLR13) as well as numerous molecules directly implicated in the signalling cascade downstream of the TLR-PAMP interaction. The immune response mediated by these PRRs implies the activation of the transcription factors NF-kappa-B, IRF3 and IRF7 [39], [40]. IRF3 and IRF7 are the main factors responsible in the induction of antiviral innate immunity by inducing the expression of type I IFN genes [41]. The expression level of these genes in the different experimental groups was represented as a heat map (Fig. 6).

DNA vaccine administration significantly up-regulated the expression of TLR8, a PRR directly implicated in the recognition of viral nucleic acids, with a fold-change (FC) of 3.5 with regard to the PBS control group at 72 h post-immunization. IRF3 and IRF7 were also affected by the vaccine at the same time point (FC = 15 and FC = 3.6, respectively). Moreover, some genes related with the activity of the transcription factor NF-kappa-B, such as the activator IKKbeta or the NF-kappa-B inhibitor zeta, were significantly down-regulated by pMCV1.4-G_860_. TLR2 recognize other viral components such as envelope glycoproteins [42] and, although no significant up-regulations were observed for this gene after vaccination, the heat map reflects a slight induction. TLR2 activation induces apoptosis through a FADD/Caspase 8 pathway [43] and both genes appeared overrepresented at 72 h, revealing a possible stimulation of the TLR pathway via TLR2 and ultimately, inducing apoptosis.

With regard to the viral challenge (PBS – VHSV and pMCV1.4– VHSV groups), it is interesting to highlight that some Toll-like receptors with a typical role in bacterial component detection (TLR5 and TLR6) have been found to be strongly regulated after VHSV infection, and this induction could suggest a novel role of these receptors in the recognition of viral components. A sequence annotated as TLR13 was also modulated at 24 h; there is little information about the function of this receptor and the nature of their ligands remains still poorly understood, but there are evidences about the recognition of bacterial rRNA [44] as well as vesicular stomatitis virus [45] by TLR13. As was expected, the typical viral-recognition receptors TLR3 and TLR8 were significantly up-regulated after VHSV challenge. Focusing the attention in the downstream signalling components, a pronounced induction of several proteins was observed (MyD88, IRF3, IRF7, FADD, Caspase-8, etc). Some molecules were also found to be down-regulated, including inhibitors of the transcription factor NF-kappa-B as well as numerous molecules implicated in their activation, possibly due to the maintenance of equilibrium in the NF-kappa-B activity.

On the other hand, VHSV infection in vaccinated fish (pMCV1.4-G_860_– VHSV) revealed a completely different pattern, even TLR2 and TLR5 were found to be significantly down-regulated at 24 h and the other TLRs were not affected by the viral infection. As a consequence, the induction of downstream proteins was practically suppressed or down-regulated and only in the case of IRF3 and IRF7 significant up-regulations were detected at 24 h, with a return to the basal levels at 72 h.

#### The IFN system

**Figure 7:**
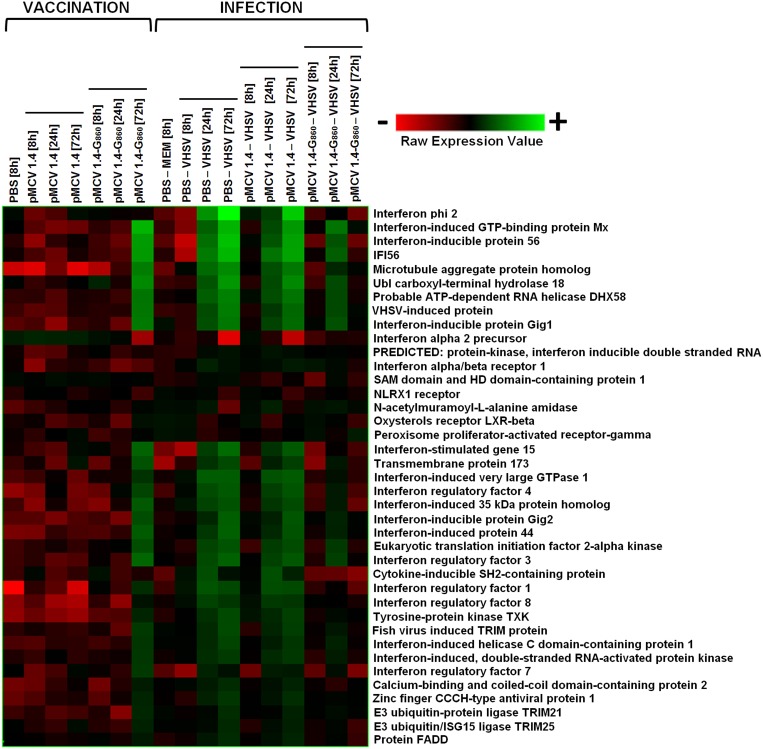
Heat map displaying hierarchical clustering results from microarray expression data of genes involved in the IFN system (IFNs, IRFs, ISGs…). All the experimental groups, including the corresponding controls (PBS 8 h and PBS – MEM 8 h), were included in the analysis. Different genes are represented in different rows, and different experiments in different columns. Raw expression values are represented as a color scale from red for lower expressions to green for higher expressions.

The interferon (IFN) system is an early antiviral immune process controlling most virus infections in the absence of specific immunity, buying time for the generation of adaptive defense mechanisms [46]. Nowadays is well known that fish type I IFNs induce the expression of a wide variety of IFN-stimulated genes (ISGs) after recognition of specific IFN receptors [47]. These ISGs reduce the viral replication and dissemination through different blocking mechanisms. Two type I IFNs, annotated as “Interferon phi 2” and “Interferon alpha 2 precursor”, as well as some of the most relevant IFN-related sequences modulated in the microarray were analyzed in the different groups (Fig. 7). Recently these two turbot type I IFNs were characterized and renamed as Ifn1 and Ifn2, respectively [48].

No up-regulations were detected in the level of any of both IFNs after vaccination, and even Interferon alpha 2 precursor (renamed as Ifn2) was significantly down-regulated in comparison with control fish (FC = −4.3). However, Interferon regulatory factors (IRFs 1, 3, 4, 7 and 8) and the majority of ISGs were up-regulated after 72 h. The most induced ISG by pMCV1.4-G_860_ was Mx (FC = 66), followed by Interferon-inducible protein 56 (FC = 44.4) and IFI56 (FC = 39.3), two genes or different parts of the same gene belonging to the IFIT (IFN-induced protein with TPR repeats) family [49]. These up-regulations in downstream genes revealed an activation of the IFN signalling pathway after immunization with pMCV1.4-G_860_ even when no up-regulations in the expression of IFNs were observed.

After viral infection a remarkable induction of these genes was observed with some exception. Thus, Interferon phi 2 (Ifn1) was highly up-regulated especially at 72 h (FC = 593.3 and FC = 177.7 in fish injected with PBS - VHSV and pMCV1.4 - VHSV, respectively), whereas Interferon alpha 2 precursor (Ifn2) transcription was down-regulated. Interestingly, we previously observed that both turbot IFNs were overexpressed after VHSV challenge although at different level, being Ifn1 strongly induced and Ifn2 slightly up-regulated and with a brief induction time [48]. Nevertheless, an overall and strong up-regulation of ISGs was observed. There are evidences suggesting that different forms of type I IFNs may have complementary antiviral activities in different cells, at different stages of infection or differ functionally [47], and this was also observed for turbot type I IFNs. Whereas Ifn1 (“Interferon phi 2”) showed a typical antiviral activity (ISGs induction and protection against VHSV infection), Ifn2 was no able to increase the expression of ISGs and therefore, did not show any protective effect against VHSV, but was able to up-regulate the level of several immune-related genes, including pro-inflammatory cytokines [48].

When vaccinated fish were infected (pMCV1.4-G_860_– VHSV), a weaker up-regulation of some IFN-related genes was observed at 24 h in comparison with both non-vaccinated groups. This modulation diminished at 72 h, nearly returning to basal levels. In fact, the high expression of Interferon phi 2 (Ifn1) after VHSV challenge was not significantly affected in pMCV1.4-G_860_ vaccinated turbot after infection at any of the sampling points. Therefore, vaccinated turbot showed a limited IFN-response after viral challenge.

#### Apoptosis

**Figure 8:**
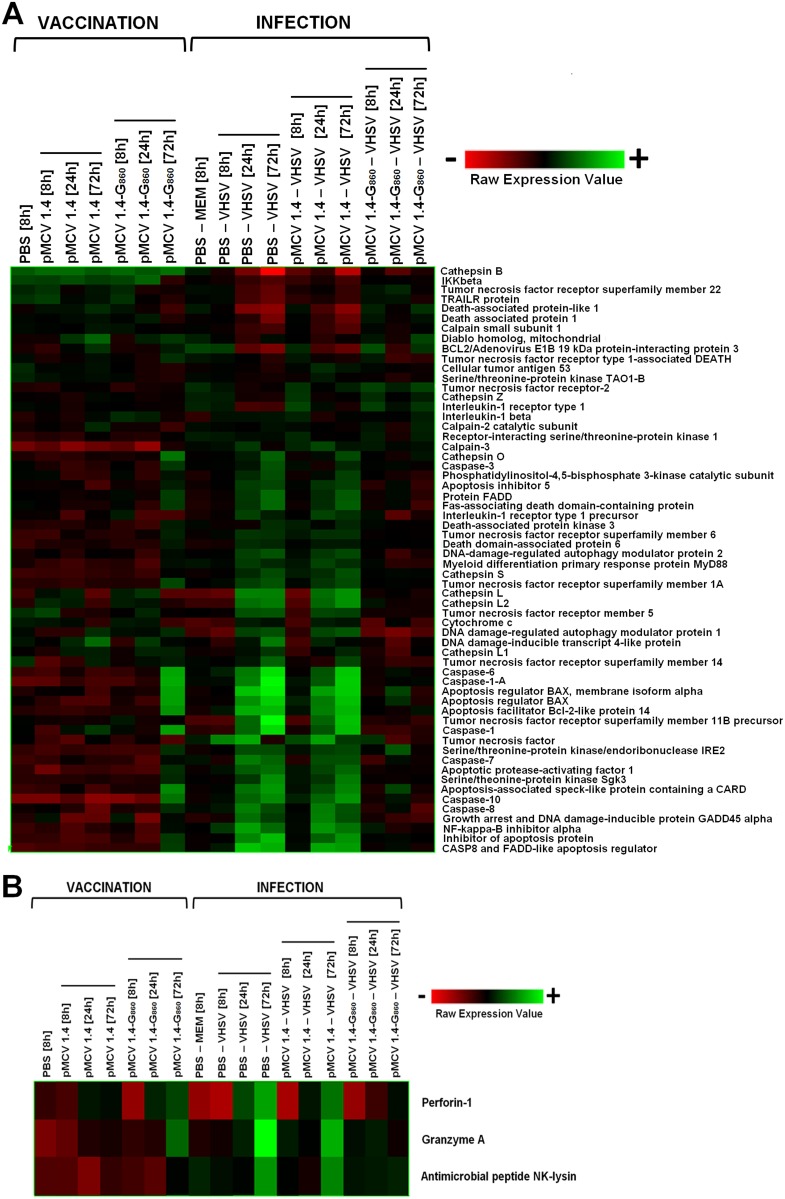
Heat map displaying hierarchical clustering results from microarray expression data of genes involved in the (A) apoptosis pathway and in the (B) cell death induced by cytotoxic cells. All the experimental groups, including the corresponding controls (PBS 8 h and PBS – MEM 8 h), were included in the analysis. Different genes are represented in different rows, and different experiments in different columns. Raw expression values are represented as a color scale from red for lower expressions to green for higher expressions.

One of the most important mechanisms preventing viral replication and dissemination is the apoptosis or programmed cell death, in which infected cells are eradicated through the activation of a group of proenzymes known as caspases [50]. The heat map containing several of the most relevant proteins implicated in apoptosis (Fig. 8A) revealed the induction of multitude of pro-apoptotic genes following pMCV1.4-G_860_ vaccination, including the initiator caspases Caspase-8 and Caspase-10 and the effector caspases Caspase-6 and Caspase-7, as well as Caspase-1 or Caspase-1A. The injection of the empty plasmid pMCV1.4 was also capable of inducing up-regulation of some genes especially implicated in the apoptotic intrinsic pathway (Diablo homolog mitochondrial, BCL2/adenovirus E1B 19 kDa protein-interacting protein 3, Calpain-2 catalytic subunit, DNA-damage-inducible transcript 4-like protein, Apoptosis regulator BAX, Cytochrome c).

VHSV infection (PBS – VHSV and pMCV1.4– VHSV groups) revealed an extensive induction of genes implicated in apoptosis. Thus, all the caspases contained in the microarray were significantly and strongly up-regulated, indicating a powerful activation of the programmed cell death. The profile reflecting the apoptotic induction after viral challenge in pMCV1.4-G_860_ vaccinated turbot was totally different. Thus, the existence of a specific immune response seems to reduce the viral transcription to a level that practically avoids the activation of the apoptotic mechanisms. Indeed, the caspases analyzed in the microarray were not affected in pMCV1.4-G_860_ vaccinated fish after VHSV challenge at any of the sampling points. Significant but slight up-regulation in the expression of some specific apoptosis genes was only detected at 24 h (Apoptosis regulator BAX, Apoptosis regulator BAX membrane isoform alpha, Apoptosis-associated speck-like protein containing a CARD).

On the other hand, cytotoxic T lymphocytes (CTLs) and natural killer (NK) cells are also able to induce cell death through the Perforin/Granzyme-induced apoptosis, in which Perforin and Granulysin (or NK-lysin) generate membrane disruption of virally infected cells and a family of structurally related serine proteases (Granzymes) induces apoptosis of the target cell activating the caspases [51]. As it is shown in Fig. 8B, at 72 h after pMCV1.4-G_860_ injection the levels of Granzyme A (FC = 7.6), Perforin-1 (FC = 3.1) and Antimicrobial peptide NK-lysin (FC = 1.8) were significantly up-regulated, indicating the activation of the cytotoxic cells after viral G glycoprotein expression. As expected, these genes mediating the cytotoxic response were also overexpressed after VHSV administration but, once again, the pattern was very different in previously vaccinated fish (pMCV1.4-G_860_– VHSV), where this effect was practically voided.

Activated cytotoxic cells induce apoptosis in virally infected cells after the recognition of MHC-I-presented peptides as foreign [52]. Moreover, cross-presentation of antigens derived from apoptotic infected cells by professional antigen presenting cells, such as dendritic cells, is another important way for initiating CD8 cytotoxic lymphocyte responses to virus [53]. Therefore, a direct correlation between apoptosis induction and overexpression of antigen presentation molecules could be established.

#### MHC class I antigen presentation

**Figure 9:**
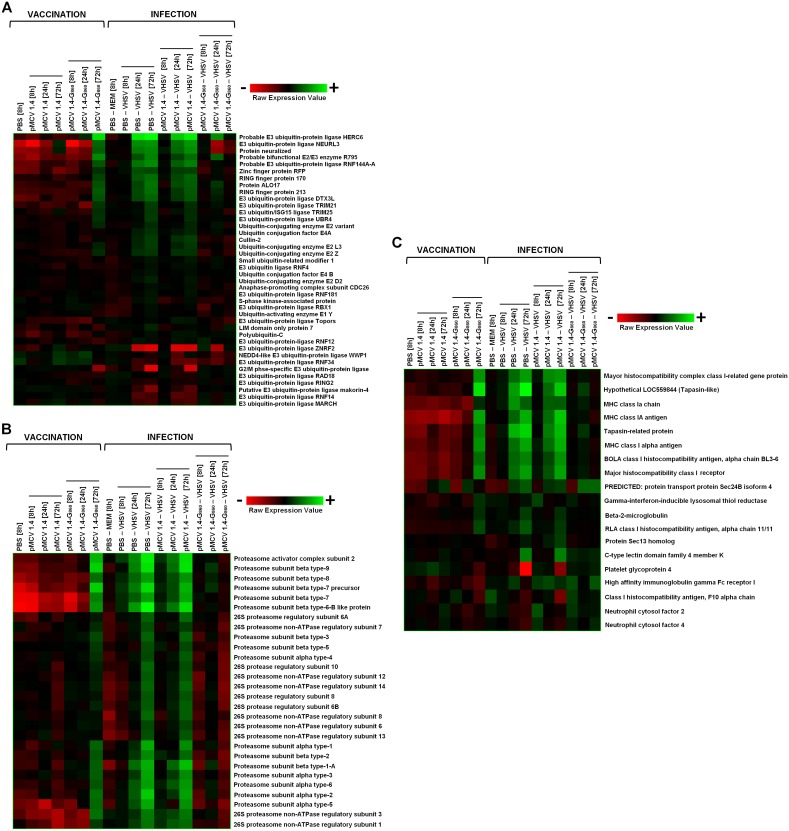
Heat map displaying hierarchical clustering results from microarray expression data of genes involved in the MHC-I antigen presentation process. (**A**) Enzymes involved in the ubiquitination of target proteins. (**B**) Subunits belonging to the proteasome complex. (**C**) MHC class I related-proteins and molecules implicated in the antigen-MHC class I assembly. All the experimental groups, including the corresponding controls (PBS 8 h and PBS – MEM 8 h), were included in the analysis. Different genes are represented in different rows, and different experiments in different columns. Raw expression values are represented as a color scale from red for lower expressions to green for higher expressions.

Protein ubiquitination is a mechanism that serves as a mark for the degradation of self and foreign proteins, such as viral molecules. The process of ubiquitination allows the recognition of proteins by the 26S proteasome, a complex that degrades ubiquitinated proteins to small peptides [54]. These peptides could be finally presented as antigens on the plasma membrane, throughout the Major Histocompatibility Complex class I (MHC-I) assembly and peptide binding process [55]. The expression pattern of several ubiquitin-related genes was analyzed for determining the effect of pMCV1.4-G_860_ vaccination as well as the response to VHSV infection (Fig. 9A). At 72 h after pMCV1.4-G_860_ vaccine injection, several genes corresponding to ubiquitin-protein ligases were up-regulated, as well as some ubiquitin-conjugating enzymes. Slight down-regulations were also observed in other ubiquitin-related genes. The viral challenge induced the expression of a broad range of these genes, but strong inhibitions of some genes were also detected and, therefore, more investigations will be necessary in order to determine the role of the different ubiquitin-related proteins in the viral antigen presentation process. On the other hand, VHSV infection of vaccinated turbot showed only a moderate induction at 24 h of some of the genes up-regulated in non-vaccinated fish and, interestingly, some ubiquitin-ligases up-regulated in both groups of non-vaccinated fish were significantly down-regulated in vaccinated individuals, especially E3 ubiquitin-protein ligase NEURL3 and Protein neuralized. The opposite response of these genes in non-immunized and immunized turbot after VHSV infection is an interesting point for further investigations.

Regarding the genes encoding the different subunits of the proteasome complex (Fig. 9B), the injection of pMCV1.4-G_860_ induced the transcription of multitude of them. More evident was the up-regulation of all the analyzed genes after viral challenge in both groups of non-vaccinated individuals, whereas vaccinated fish showed only a modest up-regulation of some of these subunits at 24 h after VHSV challenge.

As a consequence of the activation of ubiquitin and proteasome-related genes, it was expected that pMCV1.4-G_860_ administration and VHSV infection would have induced the up-regulation of the main genes implicated in the MHC-I antigen presentation (Fig. 9C). pMCV1.4-G_860_ vaccination and VHSV infection up-regulated the expression of MHC-I and related genes, although some down-regulations were also observed in genes implicated also in other biological functions (e.g. Platelet glycoprotein 4). On the other hand, turbot previously vaccinated with pMCV1.4-G_860_ presented only a modest induction of some of the above mentioned genes after viral challenge probably as consequence of the existence of an adaptive immune response limiting the proliferation success of the virus.

#### Coagulation, platelet-related proteins and complement cascade

The complement system and coagulation are two closely related pathways belonging to a complex inflammatory network and showing an intense interaction between them [56]. Pro-inflammatory cytokines play a central role in the coagulation and fibrinolysis pathways and, in an inverse way, the activation of the coagulation system may affect the inflammatory responses [57].

**Figure 10:**
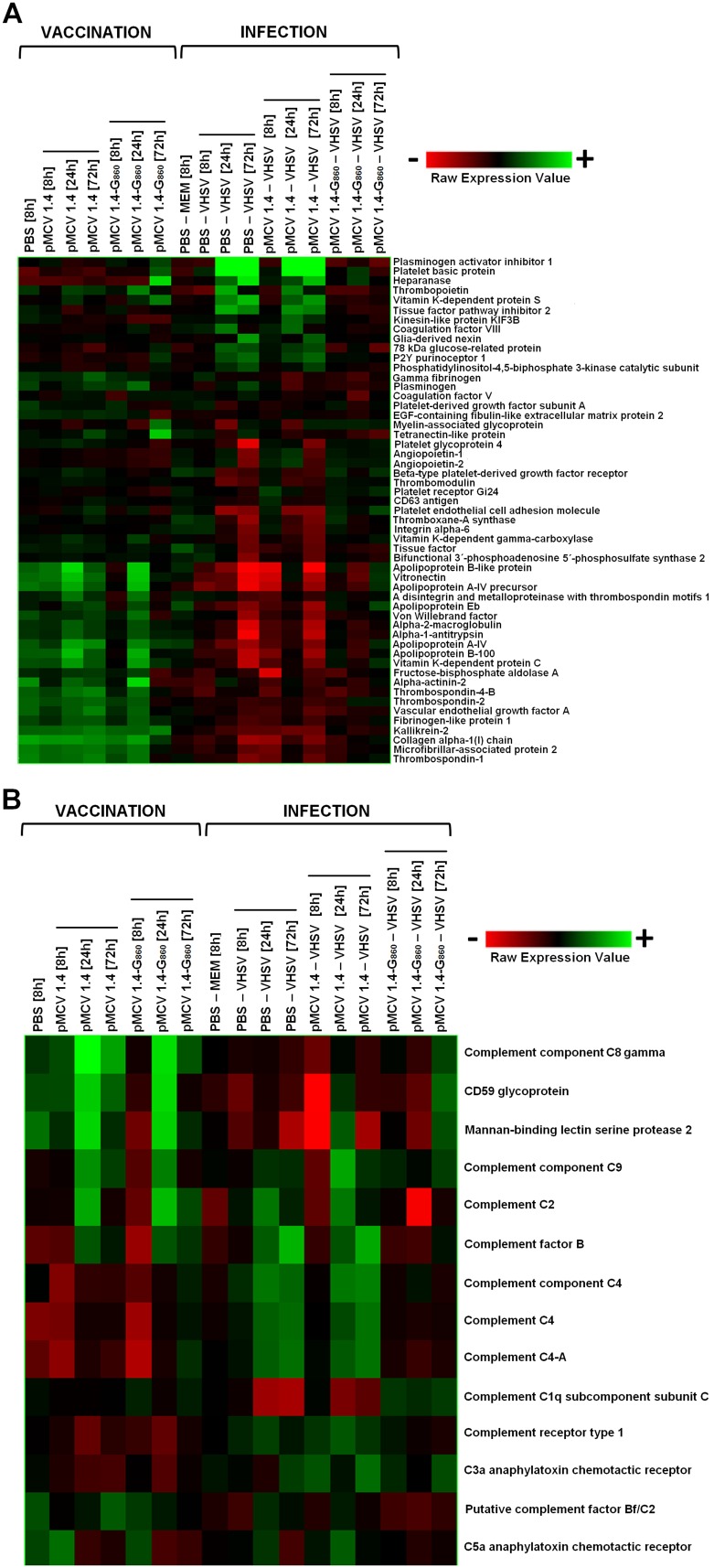
Heat map displaying hierarchical clustering results from microarray expression data of genes belonging to the (A) coagulation or (B) complement cascades. All the experimental groups, including the corresponding controls (PBS 8 h and PBS – MEM 8 h), were included in the analysis. Different genes are represented in different rows, and different experiments in different columns. Raw expression values are represented as a color scale from red for lower expressions to green for higher expressions.

Some coagulation-related genes were significantly modulated at 3 days after pMCV1.4-G_860_ vaccine administration (Fig. 10A). Thus, the most up-regulated genes were Heparanase, Tetranectin-like protein, Platelet basic protein, Vitamin K-dependent protein S and Myelin-associated protein. On the other hand, significant down-regulations were detected for example in Alpha-actinin-2, Thrombospondin-1, Thombospondin-2, EGF-containing fibulin-like extracellular matrix protein 2 and von Willebrand factor among others. Both coagulant and anticoagulant genes were up and down-regulated after pMCV1.4-G_860_ vaccine injection and, therefore, it is difficult to establish a general pattern with regard to this process.

After VHSV infection an intense regulation of these genes was also observed. Thus, some anticoagulatory and procoagulatory genes were tightly up-regulated but others were down-regulated. Viral Haemorrhagic Septicaemia (VHS) is a viral disease causing widespread haemorrhages (bleeding) in fish tissues, including internal organs. It is well known that tissue injury induces an inflammatory response and the systemic inflammation is a potent prothombotic stimulus, up-regulating procoagulant factors, down-regulating natural anticoagulants and inhibiting fibrinolytic activity [58]. Although the role of inflammation is to resolve infection and injury, excessive or altered inflammation often leads to a wide range of tissue injuries and diseases, such as multi-organ failure [59]. Therefore, the equilibrium between pro-inflammatory and anti-inflammatory molecules is essential for the host survival. This could be the explanation for the alternative modulation of these coagulation-related proteins during VHSV infection. As occurs with the previously analyzed groups of immune-related proteins, the VHSV infection of pMCV1.4-G_860_ vaccinated turbot showed a more moderate modulation of these genes. The presence of specific immunity would lead to a decrease of tissue injury and inflammation through the reduction of the number of viral particles in the fish.

As mentioned above, the complement system is a cascade closely related with the coagulation, with numerous interactions between both pathways. Indeed, thrombin acts as a potent C3 and C5 convertase, leading the generation of the anaphylotoxins C3a and C5a [56]. The central component of the complement system is the component C3, which is proteolytically activated through the classical, lectins and alternative routes [60], as well as by the coagulation system. As it is observed in Fig. 10B, the administration of the plasmid (both pMCV1.4 and pMCV1.4-G_860_) up-regulated some complement components, especially at 24 h (for instance, Complement component C8 gamma, CD59 glycoprotein, Mannan-binding lectin serine protease 2, Complement component C9, Complement C2, Complement factor B and Complement C4). In contrast, Complement receptor type 1, C3a anaphylatoxin chemotactic receptor and C5a anaphylatoxin chemotactic receptor were down-regulated 24 h after any plasmid injection. Therefore, the plasmid DNA backbone was able to induce an immune response at the complement level. The modulation in the expression of these genes could be related to the differences observed between both groups of non-vaccinated fish after viral challenge (higher number of affected genes at 8 h post-infection, lower transcription of viral genes and reduction and delay in the mortality of fish previously injected with pMCV1.4). In addition, pMCV1.4-G_860_ vaccination up-regulated the expression of Complement C4 and Complement C4-A, and down-regulated the level of C5a anaphylatoxin chemotactic receptor at 72 h.

On the other hand, VHSV challenge increased the expression of the majority of the analyzed genes but, interestingly, a significant reduction in the transcription of Complement C1q subcomponent subunit C was observed. However, this transcript appeared slightly up-regulated in pMCV1.4-G_860_– VHSV turbot at 8, 24 and 72 h after infection. C1 complex is part of the classical pathway of the complement, which implicates the participation of antibodies. The presence of specific antibodies against VHSV in pMCV1.4-G_860_ vaccinated fish could be affecting the expression of Complement C1q subcomponent subunit C and therefore, favouring the classical pathway of the complement system.

#### Markers of activation and proliferation of immune-relevant cell types

**Figure 11:**
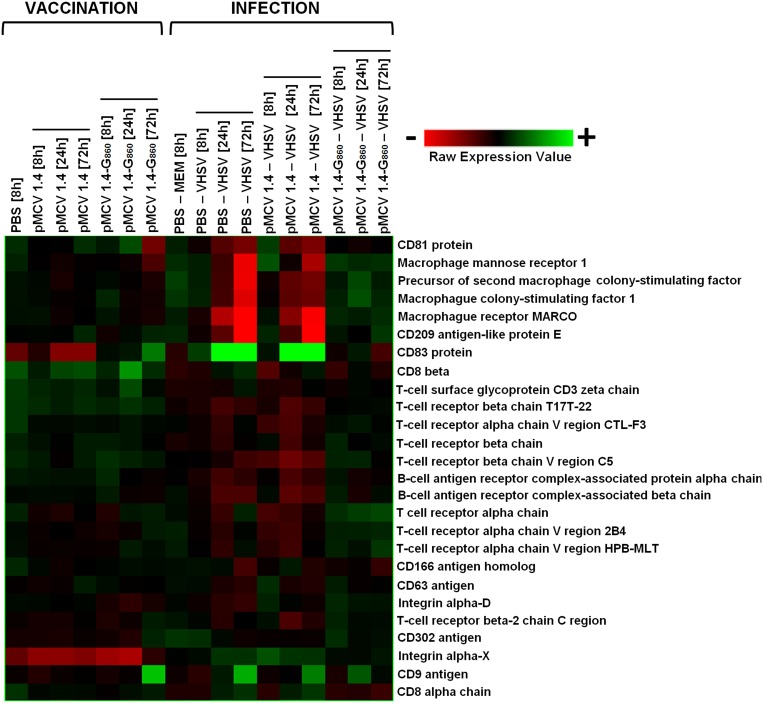
Heat map displaying hierarchical clustering results from microarray expression data of gene markers of activation and proliferation of cell types. All the experimental groups, including the corresponding controls (PBS 8 h and PBS – MEM 8 h), were included in the analysis. Different genes are represented in different rows, and different experiments in different columns. Raw expression values are represented as a color scale from red for lower expressions to green for higher expressions.

The last hierarchical analysis was conducted for determining the modulation of genes implicated in the maturation, proliferation or activation of different cell lineages. Some membrane markers and two sequences with homology to macrophage colony-stimulating factors were selected and their pattern of relative expression obtained (Fig. 11). Three days after vaccination down-regulations were observed for the gene corresponding to the CD81 protein (FC = −3.6) and the Macrophage mannose receptor 1 (FC = −2.5). CD81 is a tetraspanin cell surface protein showing a broad expression on numerous immune cells and it is known to play an important role in multiple cellular interactions [61]. Chang et al. [62] found that interferon-alpha treatment is able to suppress the CD81 expression, possibly through the activity of the double-stranded RNA activated kinase; our results revealed a significant up-regulation of this ISG after pMCV1.4-G_860_ vaccination in turbot. The other down-regulated gene, Macrophage mannose receptor 1, is a cell surface transmembrane glycoprotein expressed on macrophages that serves as a phagocytic receptor mediating the binding and ingestion microorganisms with a mannose-rich surface [63]. Harris et al. [64] observed that IFN-γ administration decreased-to-absent the cell-surface mannose receptor transcription in murine macrophages. There are not sequences homologues to IFN-γ in the turbot microarray and therefore, a negative correlation between the two proteins cannot be established. However, Purcell et al. [14] found a high up-regulation of IFN-γ in rainbow trout in the site of injection (muscle) 7 days after vaccination against the IHNV rhabdovirus after injecting a DNA vaccine encoding its G glycoprotein. CD209 antigen-like protein E, CD83 and CD9 were significantly up-regulated after vaccination. These three clusters of differentiation were also induced in rainbow trout one week after injection of a similar DNA vaccine from IHNV [14]. CD209 antigen-like protein E and CD83 are mainly expressed in dendritic cells and these up-regulations could be indicating a proliferation and activation of this cell type, which forms a system of professional antigen-presenting cells.

VHSV infection especially induced the up-regulation of the gene encoding the CD83 protein, indicating a possible activation/proliferation of dendritic cells. Interestingly, this dendritic cells marker was also found to be induced on monocytes [65] and polymorphonuclear neutrophils [66] under the influence of specific cytokines, such as TNF-α, acquiring characteristics of dendritic cells. Slight up-regulations were also observed for the CD166 antigen homolog, CD9 as well as for CD8 alpha chain and CD8 beta, reflecting a putative activation of CD8^+^ T lymphocytes (cytotoxic cells). However, weak down-regulations were also obtained for several T-cell receptor (TCR) chain regions (fold-changes between −1.5 and −2 in both non-vaccinated groups). B-cell receptor complex-associated protein alpha chain and B-cell receptor complex-associated protein beta chain (BCR chains) were also down-modulated with a fold-change around −2. But the more surprising down-regulations were observed for some molecules with an exclusive/closely relation with macrophages. Thus, the Macrophage receptor MARCO and the Macrophage mannose receptor 1, two phagocyte receptor molecules serving as a pattern-recognition receptor for bacterial components, were highly down-modulated by VHSV administration especially after 72 h. In PBS - VHSV fish, MARCO showed a FC = −46 and Macrophage mannose receptor 1 a FC = −10.2, whereas in pMCV 1.4 - VHSV turbot showed FC = −10.2 and −5.7, respectively. Taking this information into consideration, together with the strong down-regulation of Macrophage colony-stimulating factor 1 and Precursor of second macrophage colony-stimulating factor, a reduction in the macrophage proliferation or differentiation could be happening. An alternative explanation would be a massive infection and destruction of these cells by the virus. It has been shown that VHSV is able to infect turbot blood leukocytes and kidney macrophages [67]. However, further investigations have reported that macrophages are heterogeneous in their permissiveness for viral penetration and usually only a subpopulation is infected [67]–[70]. Therefore, the drastic effect induced by VHSV in the expression of macrophage-related proteins is still poorly understood and more studies need to be performed for understanding if VHSV massively replicates in this cell type. It is also interesting to highlight that Interleukin-18 (IL-18) was down-regulated 72 h after VHSV challenge. IL-18 is a potent pro-inflammatory cytokine essential to host defenses against severe infections, taking part in the clearance of viruses [71]. Macrophages are the main IL-18 producers in response to stimuli of viral/bacterial origin [72] and the reduction in the mRNA level of this cytokine after VHSV challenge was probably related with the inhibition of the macrophage markers described above.

Little changes in the expression of the selected proteins were observed in vaccinated turbot after infection, but a slight tendency toward TCR sequences up-regulation was observed. Purcell et al. [14] also suggested an enhanced T cell activation or proliferation serving as mechanism of protection in Japanese flounder immunized using a DNA vaccine encoding the HIRRV G gene when fish were infected with the virus.

## Conclusions

In summary, we have analyzed the importance of different immune processes implicated in the protection provided by pMCV1.4-G_860_ in turbot before and after VHSV infection. This work represents the most exhaustive transcriptomic study about DNA vaccines against rhabdoviral pathogens in fish. The high-throughput screening provided will serve as a basis for a better understanding of the molecular processes implicated in the successful vaccination protocols against viral diseases in fish. The plasmid pMCV1.4-G_860_ induces a powerful immune response at 3 days after vaccination affecting the main immune processes and leading to an efficient antigen presentation and production of specific antibodies. After VHSV challenge, non-vaccinated fish revealed an uncontrolled immune response generating an intense pro-inflammatory status in the host. In contrast, vaccinated fish showed a moderate and controlled response due to the previous presence of specific immune factors. The analyses performed in this work provide interesting information about molecules with a potential use as vaccine adjuvants, antiviral treatments or markers for vaccine efficiency monitoring as well. Moreover, some clues about the infectivity mechanisms of VHSV in fish are also proposed.

## Supporting Information

Figure S1 **Gene Ontology (GO) assignment (2^nd^ level biological process terms) of sequences modulated in head kidney at 8, 24 and 72 h after pMCV1.4 or pMCV1.4-G_860_ injection.**
(PDF)

Figure S2 **Venn diagrams reflecting the number of exclusive and common up- and down-regulated genes after pMCV1.4 and pMCV1.4-G_860_ administration.**
(PDF)

Figure S3 **Biological process multilevel pie chart reflecting exclusive and common genes at 72 h after pMCV1.4 or pMCV1.4-G_860_ injection.**
(PDF)

Figure S4 **Gene Ontology (GO) assignment (2^nd^ level biological process terms) of sequences modulated in head kidney at 8, 24 and 72 h after VHSV infection in vaccinated (pMCV1.4-G_860_ - VHSV) and non-vaccinated (PBS – VHSV and pMCV1.4– VHSV) turbot.**
(PDF)

Figure S5 **Venn diagrams reflecting the number of exclusive and common up- and down-regulated genes in PBS – VHSV, pMCV 1.4 - VHSV and pMCV1.4-G_860_– VHSV groups.**
(PDF)

Table S1 **List of primers used for qPCR validation of the microarray data.**
(DOCX)

Table S2 **Microarray and qPCR values obtained for the 5 selected immune-related genes in the microarray validation.**
(DOCX)
