# Supplementary figures and images for: Transcriptome Profiles Associated to VHSV Infection or DNA Vaccination in Turbot (Scophthalmus maximus)
Source: PLoS One. 2014 Aug 6;9(8):e104509. doi: 10.1371/journal.pone.0104509 (PMC4123995; doi:10.1371/journal.pone.0104509)

pMCV 1.4

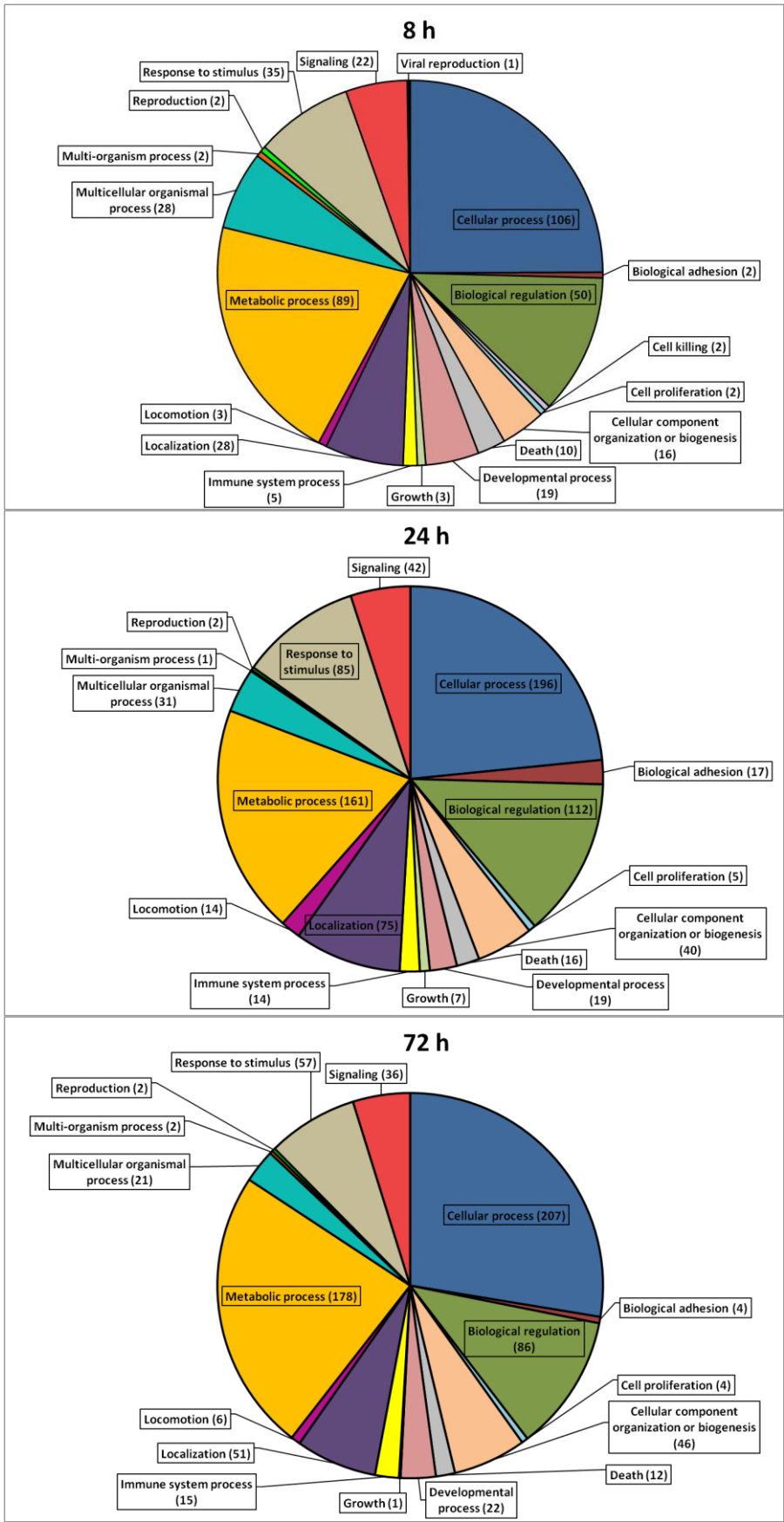

pMCV 1.4-G860

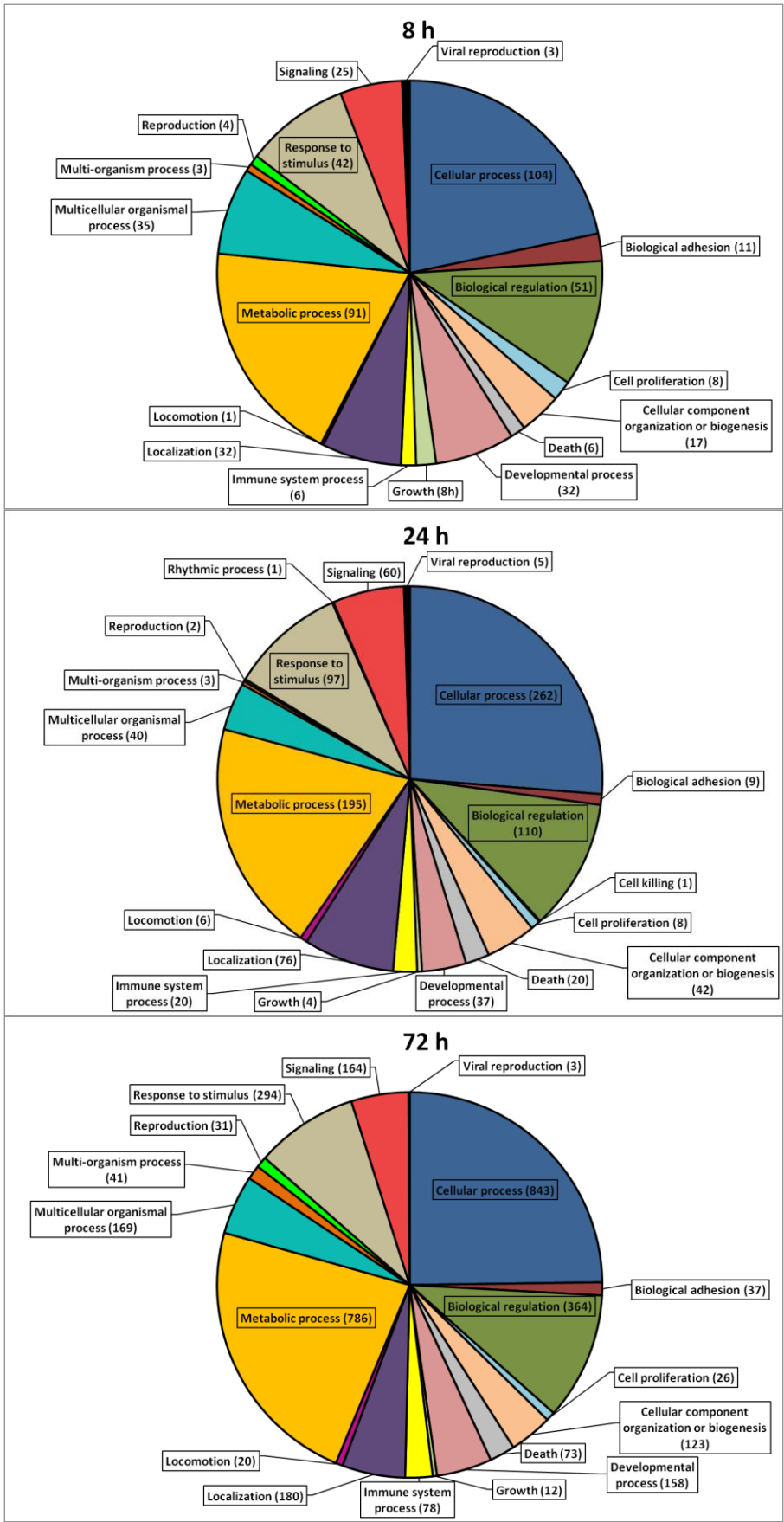

Supplement: Figure S1 — Gene Ontology (GO) assignment (2nd level biological process terms) of sequences modulated in head kidney at 8, 24 and 72 h after pMCV1.4 or pMCV1.4-G860 injection. (PDF) [file pone.0104509.s001.pdf]

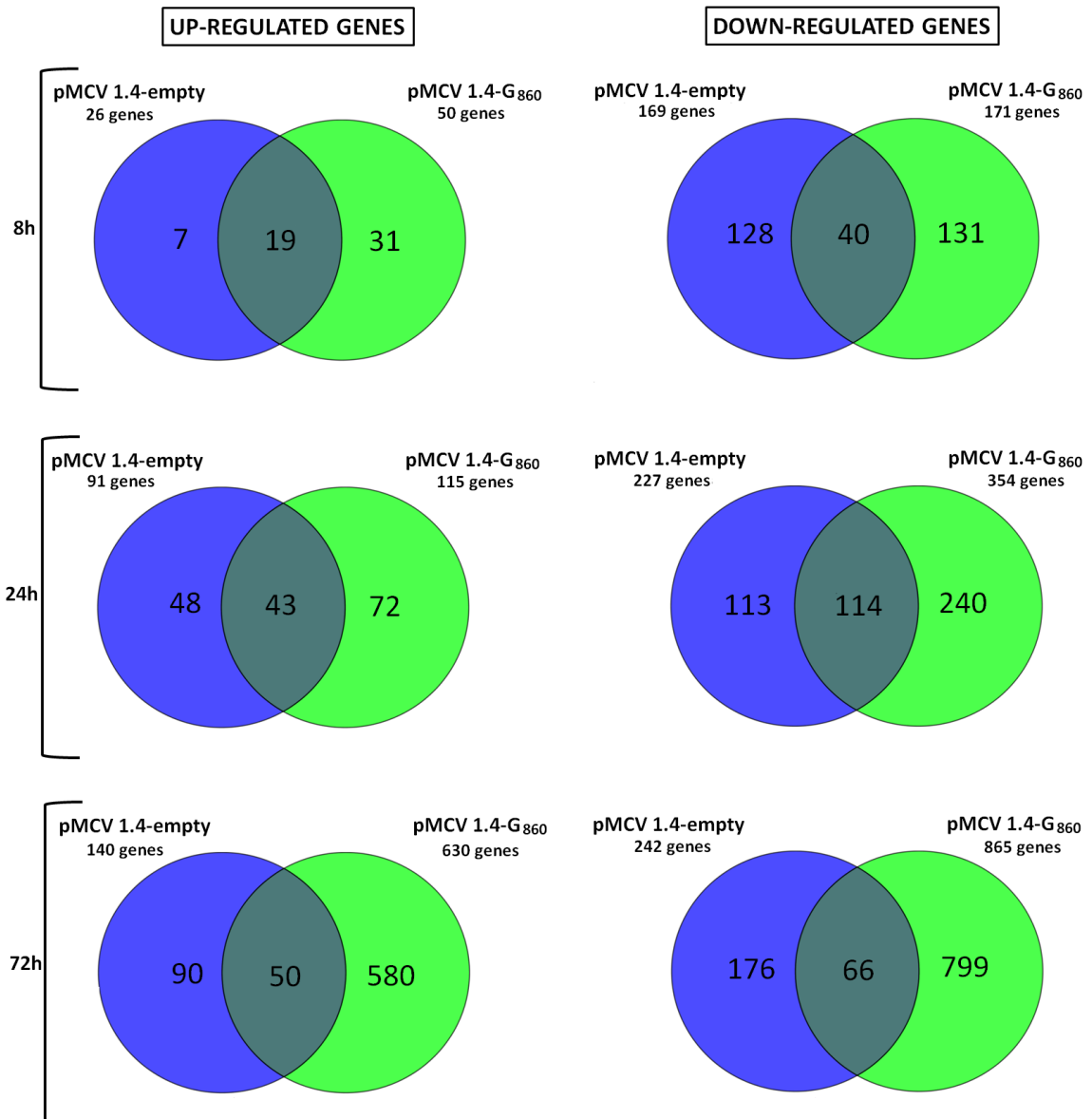

Supplement: Figure S2 — Venn diagrams reflecting the number of exclusive and common up- and down-regulated genes after pMCV1.4 and pMCV1.4-G860 administration. (PDF) [file pone.0104509.s002.pdf]

PBS - VHSV

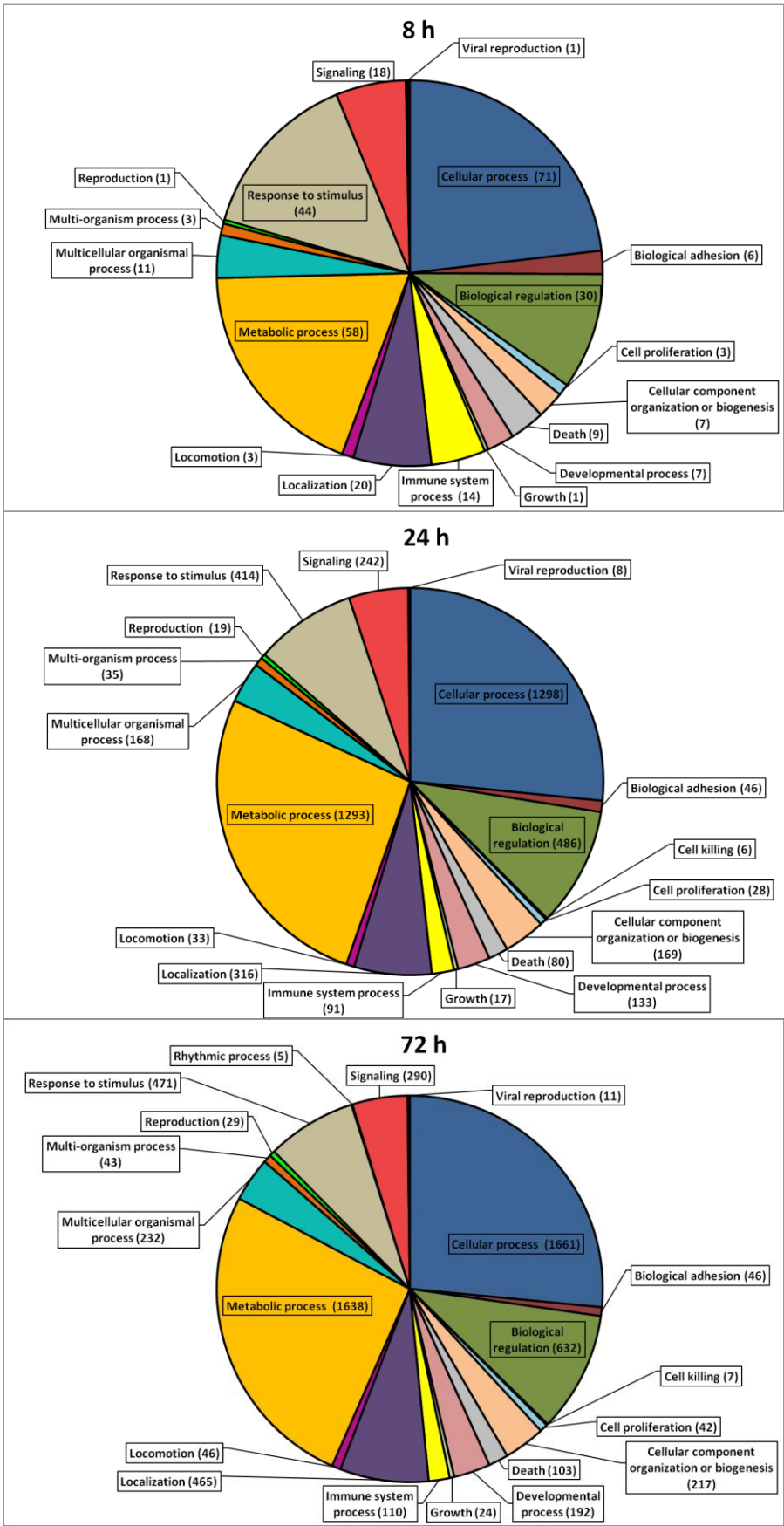

pMCV 1.4 - VHSV

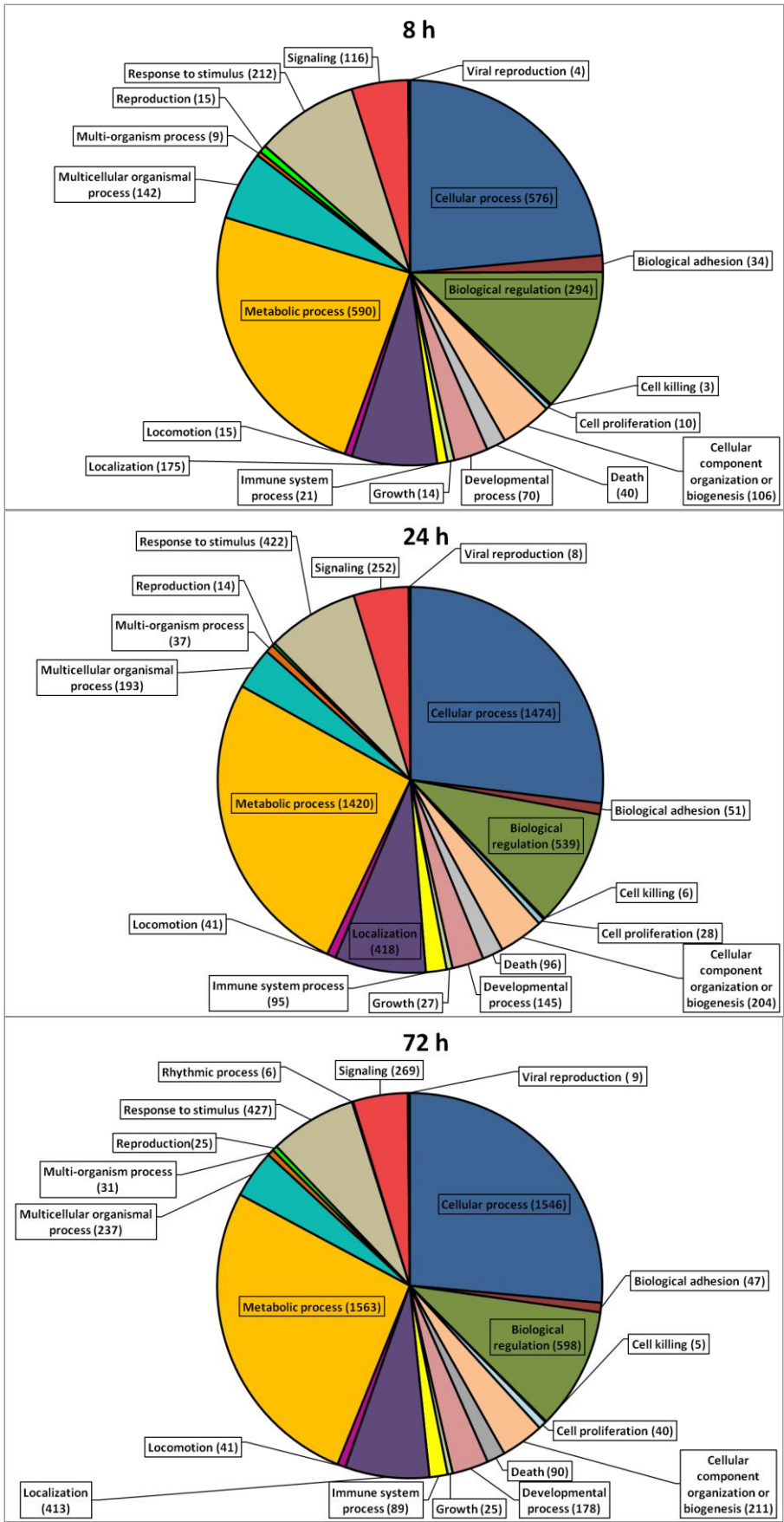

pMCV 1.4-G<sub>860</sub> - VHSV

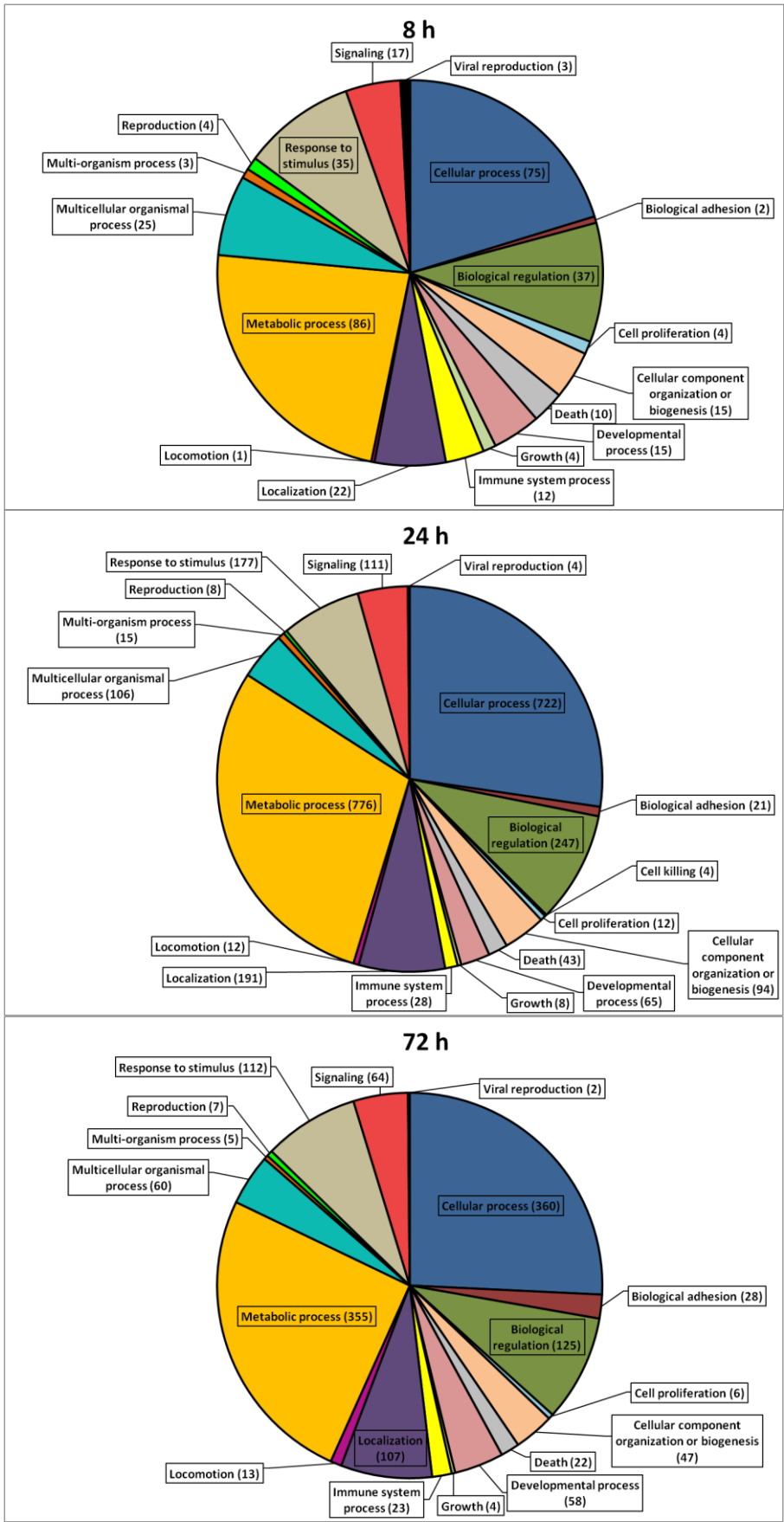

Supplement: Figure S4 — Gene Ontology (GO) assignment (2nd level biological process terms) of sequences modulated in head kidney at 8, 24 and 72 h after VHSV infection in vaccinated (pMCV1.4-G860 - VHSV) and non-vaccinated (PBS – VHSV and pMCV1.4– VHSV) turbot. (PDF) [file pone.0104509.s004.pdf]

### UP-REGULATED GENES

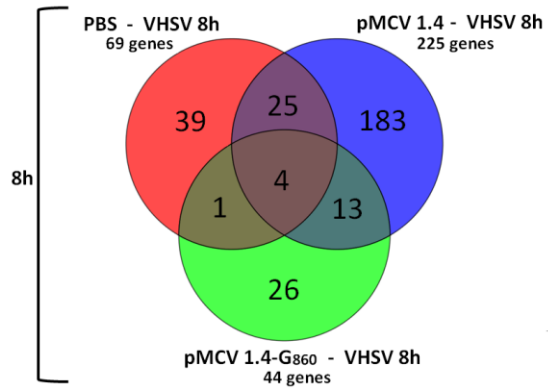

### DOWN-REGULATED GENES

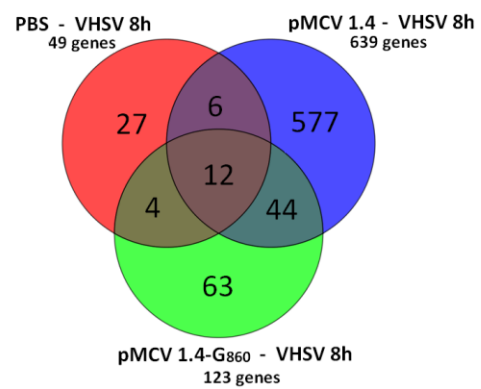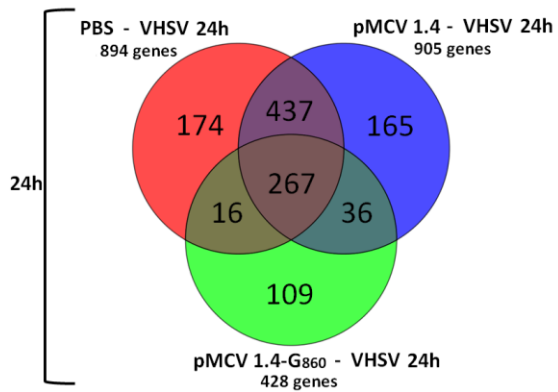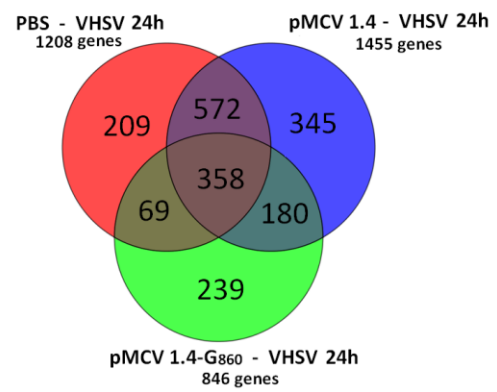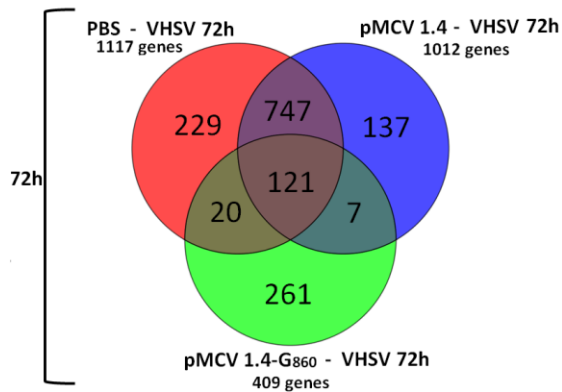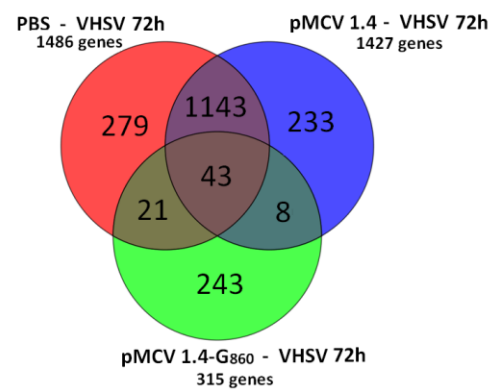

Supplement: Figure S5 — Venn diagrams reflecting the number of exclusive and common up- and down-regulated genes in PBS – VHSV, pMCV 1.4 - VHSV and pMCV1.4-G860– VHSV groups. (PDF) [file pone.0104509.s005.pdf]
