# Supplementary material for: Transcriptome Profiles Associated to VHSV Infection or DNA Vaccination in Turbot (Scophthalmus maximus)
Source: PLoS One. 2014 Aug 6;9(8):e104509. doi: 10.1371/journal.pone.0104509 (PMC4123995; doi:10.1371/journal.pone.0104509)

Exclusive regulated sequences at 72 h after pMCV 1.4-G_{860} immunisation

Up-regulated

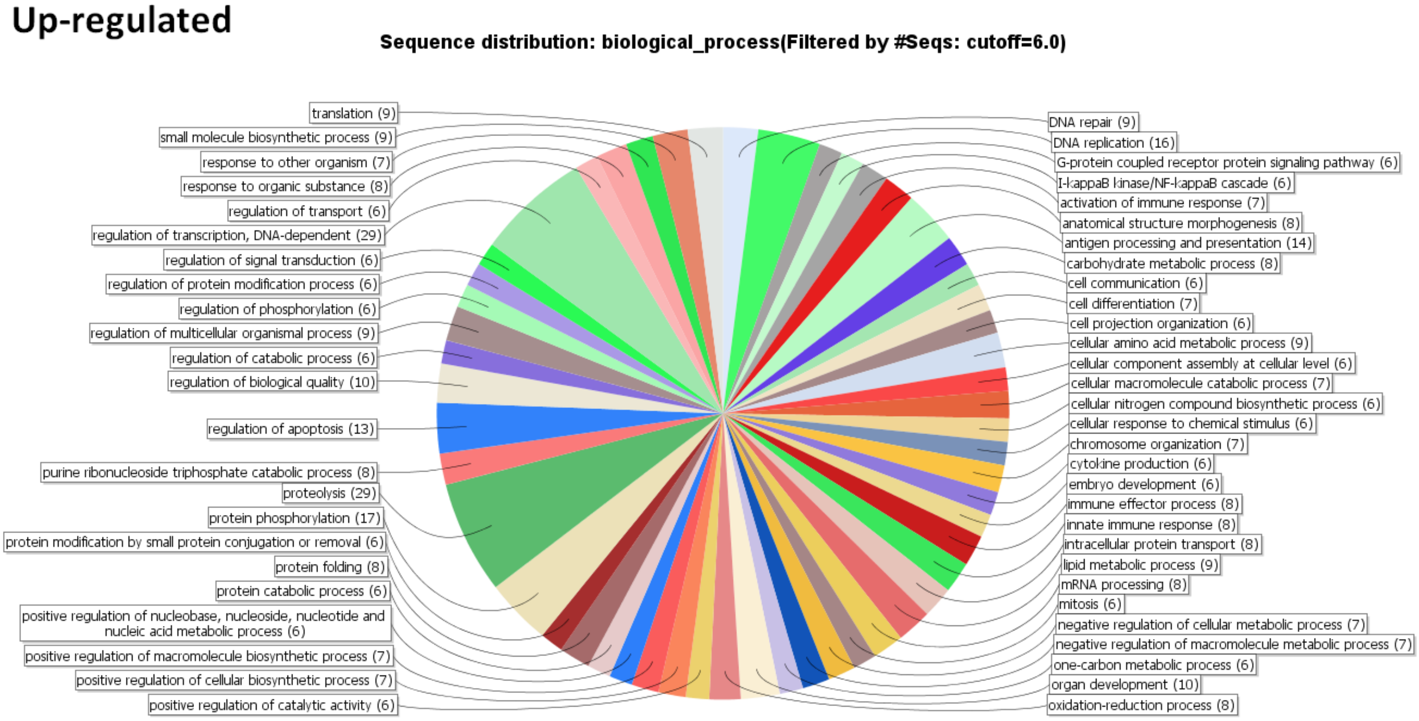

Down-regulated

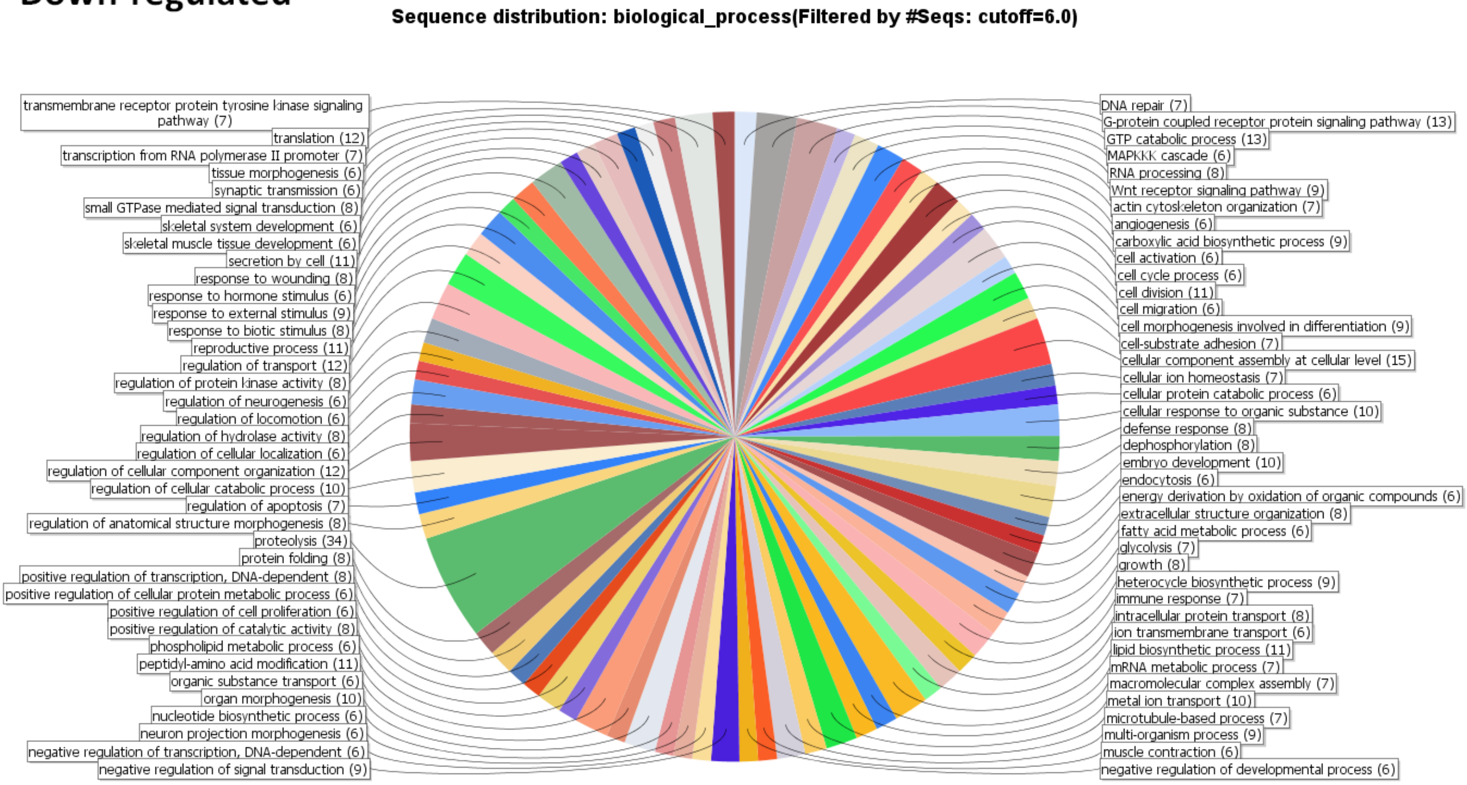

Exclusive regulated sequences at 72 h after pMCV 1.4 immunisation

Up-regulated

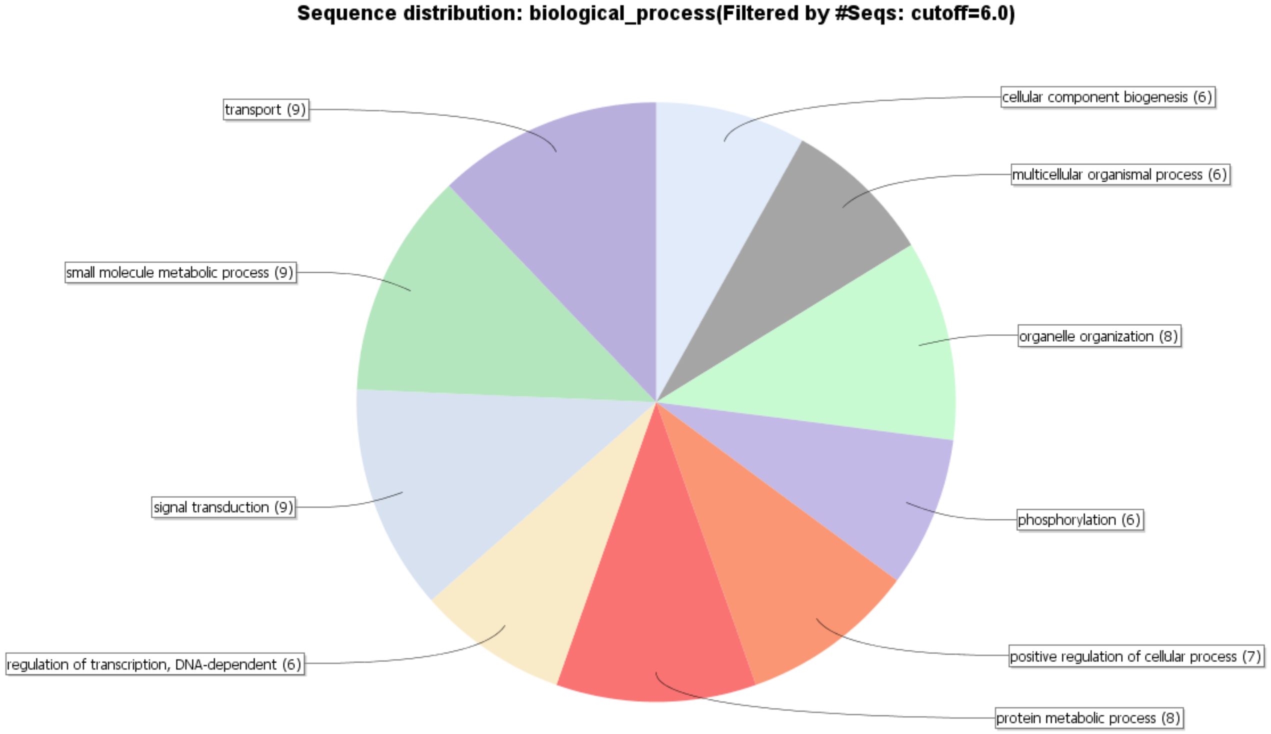

Down-regulated

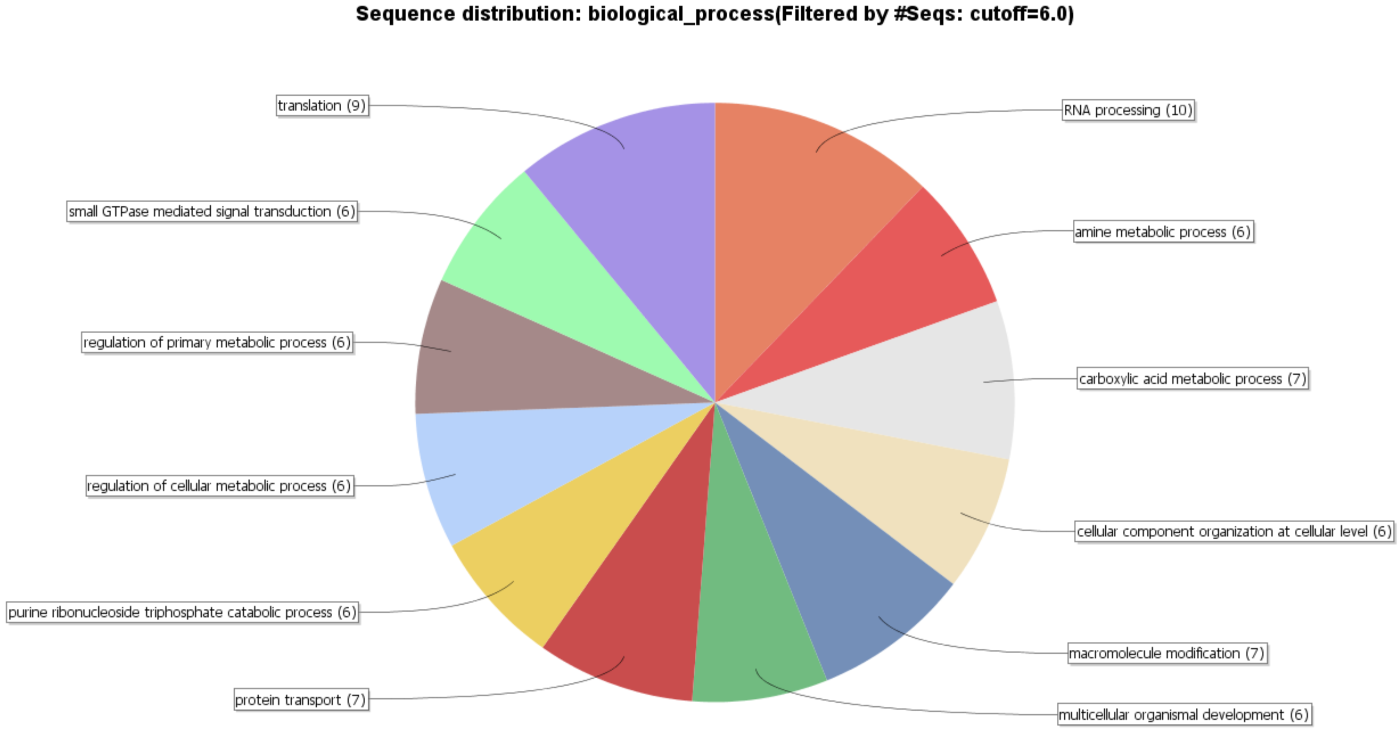

Common regulated sequences at 72 h after pMCV 1.4 or pMCV 1.4-G_{860} immunisation

Up-regulated

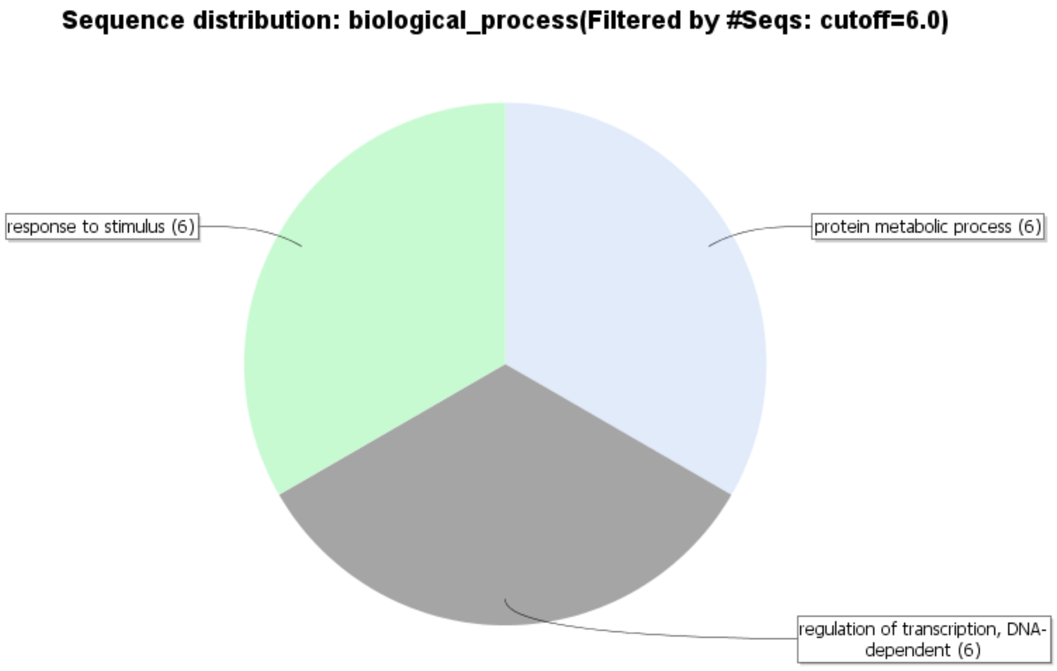

Down-regulated

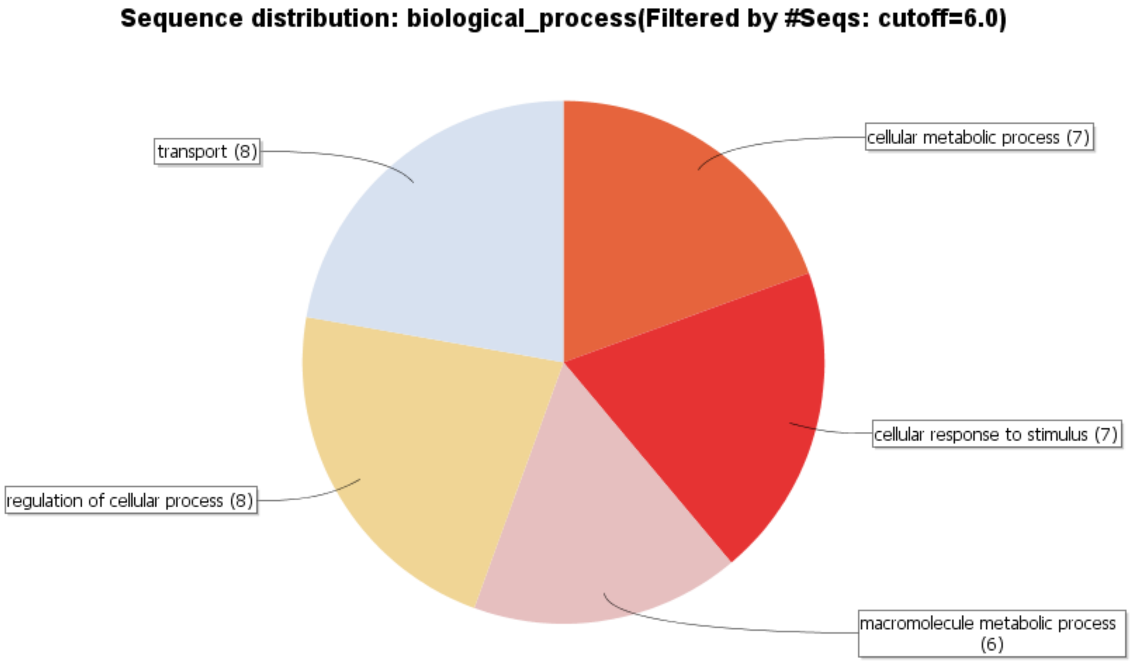

Supplement: Figure S3 — Biological process multilevel pie chart reflecting exclusive and common genes at 72 h after pMCV1.4 or pMCV1.4-G860 injection. (PDF) [file pone.0104509.s003.pdf]
