# Supplementary material for: Transcriptome Profiles Associated to VHSV Infection or DNA Vaccination in Turbot (Scophthalmus maximus)
Source: PLoS One. 2014 Aug 6;9(8):e104509. doi: 10.1371/journal.pone.0104509 (PMC4123995; doi:10.1371/journal.pone.0104509)
Supplement: Table S1 — List of primers used for qPCR validation of the microarray data. (DOCX) [file pone.0104509.s006.docx]

**Table S1-** List of primers used for qPCR validation of the microarray data.

|  | **Forward** | **Reverse** |
| --- | --- | --- |
| **Elongation factor 1-alpha** | GGAGGCCAGCTCAAAGATGG | ACAGTTCCAATACCGCCGATTT |
| **Tumor necrosis factor** | GGACAGCCACAGAGATGGAC | GCAAACACACCGAAGAAGGT |
| **Interferon phi 2** | TGTCTGTCCACAGTCAAAGGT | GGTCTTCAGGACGGAGAGG |
| **Mx** | CTGCCAGATGCTTCAGGATA | TGCAACCAATGTCCAAGTTC |
| **IFI56** | CAGCTGAACCAGAAACAGAGAA | CAGGGTTATTTCACTCTGAGCA |
| **ISG15** | CACCTATGACATCACACCAGAA | GACGTGGTAGTCCGACAGTTTA |
