# Supplementary material for: Transcriptome Profiles Associated to VHSV Infection or DNA Vaccination in Turbot (Scophthalmus maximus)
Source: PLoS One. 2014 Aug 6;9(8):e104509. doi: 10.1371/journal.pone.0104509 (PMC4123995; doi:10.1371/journal.pone.0104509)
Supplement: Table S2 — Microarray and qPCR values obtained for the 5 selected immune-related genes in the microarray validation. (DOCX) [file pone.0104509.s007.docx]

**Table S2.** Microarray and qPCR values obtained for the 5 selected immune-releted genes in the microarray validation.

|  |  |  | **Fold-change** | |
| --- | --- | --- | --- | --- |
|  |  |  | **qPCR** | **Microarray** |
| **Tumor necrosis factor** | **pMCV 1.4** | **8 h** | -1,23 | -1,56 |
|  |  | **24 h** | -1,21 | -1,86 |
|  |  | **72 h** | -1,60 | -1,73 |
|  | **pMCV 1.4-G860** | **8 h** | 1,36 | -1,25 |
|  |  | **24 h** | -1,14 | -1,48 |
|  |  | **72 h** | 146,60 | 65,98 |
|  | **PBS - VHSV** | **8 h** | 4,95 | 2,62 |
|  |  | **24 h** | 9,55 | 3,68 |
|  |  | **72 h** | -1,34 | -1,55 |
|  | **pMCV 1.4 - VHSV** | **8 h** | 3,91 | 1,51 |
|  |  | **24 h** | 6,35 | 3,42 |
|  |  | **72 h** | 1,04 | 1,34 |
|  | **pMCV 1.4-G860 - VHSV** | **8 h** | -1,03 | 1,23 |
|  |  | **24 h** | -1,99 | -2,05 |
|  |  | **72 h** | -1,92 | -1,56 |
| **Interferon phi 2** | **pMCV 1.4** | **8 h** | -1,17 | -2,16 |
|  |  | **24 h** | 1,59 | -1,85 |
|  |  | **72 h** | -1,12 | 1,05 |
|  | **pMCV 1.4-G860** | **8 h** | 1,53 | -1,11 |
|  |  | **24 h** | 1,06 | -1,30 |
|  |  | **72 h** | 16,86 | -1,33 |
|  | **PBS - VHSV** | **8 h** | -1,06 | -1,29 |
|  |  | **24 h** | 361,70 | 46,76 |
|  |  | **72 h** | 20746,71 | 593,27 |
|  | **pMCV 1.4 - VHSV** | **8 h** | 1,69 | 2,69 |
|  |  | **24 h** | 156,62 | 6,59 |
|  |  | **72 h** | 6907,88 | 177,71 |
|  | **pMCV 1.4-G860 - VHSV** | **8 h** | 1,13 | 1,11 |
|  |  | **24 h** | 3,15 | 1,61 |
|  |  | **72 h** | -1,61 | -1,11 |
| **Mx** | **pMCV 1.4** | **8 h** | -1,23 | -1,56 |
|  |  | **24 h** | -1,21 | -1,86 |
|  |  | **72 h** | -1,60 | -1,73 |
|  | **pMCV 1.4-G860** | **8 h** | 1,36 | -1,25 |
|  |  | **24 h** | -1,14 | -1,48 |
|  |  | **72 h** | 146,60 | 65,98 |
|  | **PBS - VHSV** | **8 h** | -1,32 | -1,46 |
|  |  | **24 h** | 14,51 | 12,26 |
|  |  | **72 h** | 107,14 | 88,27 |
|  | **pMCV 1.4 - VHSV** | **8 h** | -1,90 | 1,09 |
|  |  | **24 h** | 8,46 | 7,19 |
|  |  | **72 h** | 103,25 | 45,13 |
|  | **pMCV 1.4-G860 - VHSV** | **8 h** | -1,37 | 1,28 |
|  |  | **24 h** | 19,67 | 19,15 |
|  |  | **72 h** | 1,19 | 1,74 |
| **IFI56** | **pMCV 1.4** | **8 h** | -1,26 | -1,34 |
|  |  | **24 h** | -1,89 | -1,61 |
|  |  | **72 h** | -1,26 | -1,03 |
|  | **pMCV 1.4-G860** | **8 h** | 1,07 | -1,25 |
|  |  | **24 h** | -1,73 | -1,37 |
|  |  | **72 h** | 43,88 | 39,29 |
|  | **PBS - VHSV** | **8 h** | 1,46 | -2,04 |
|  |  | **24 h** | 48,95 | 20,91 |
|  |  | **72 h** | 200,05 | 86,37 |
|  | **pMCV 1.4 - VHSV** | **8 h** | -1,00 | 1,13 |
|  |  | **24 h** | 32,17 | 17,77 |
|  |  | **72 h** | 142,78 | 53,55 |
|  | **pMCV 1.4-G860 - VHSV** | **8 h** | 1,11 | 1,14 |
|  |  | **24 h** | 21,12 | 10,89 |
|  |  | **72 h** | 1,61 | -1,06 |
| **Interferon-stimulated gene 15** | **pMCV 1.4** | **8 h** | -1,00 | -1,28 |
|  |  | **24 h** | 1,02 | -1,41 |
|  |  | **72 h** | -1,10 | 1,38 |
|  | **pMCV 1.4-G860** | **8 h** | 1,83 | 1,23 |
|  |  | **24 h** | 1,32 | -1,12 |
|  |  | **72 h** | 207,34 | 10,72 |
|  | **PBS - VHSV** | **8 h** | 1,75 | -1,32 |
|  |  | **24 h** | 58,00 | 7,49 |
|  |  | **72 h** | 259,51 | 21,92 |
|  | **pMCV 1.4 - VHSV** | **8 h** | -1,21 | 1,52 |
|  |  | **24 h** | 44,83 | 5,76 |
|  |  | **72 h** | 168,02 | 14,64 |
|  | **pMCV 1.4-G860 - VHSV** | **8 h** | -1,25 | -1,01 |
|  |  | **24 h** | 17,06 | 1,78 |
|  |  | **72 h** | 1,68 | 1,28 |
